# London Trauma Conference 2024

**DOI:** 10.1186/s13049-024-01278-y

**Published:** 2024-12-09

## Oral Presentations

### O1. A novel implementation of the REDS score: an evaluation of the REDS score in mortality prediction in adult major trauma patients

#### Tirion Rhys Bonta^1^, Narani Sivayoham^2^, Harriet Tucker^2^

##### ^1^St George’s University of London, London, UK; ^2^Emergency Department, St George’s NHS Foundation Trust, London, UK

**Correspondence:** Tirion Rhys Bonta (m2103062@sgul.ac.uk)

*Scandinavian Journal of Trauma, Resuscitation and Emergency Medicine* 2024, **32(S1)**: O1

**Background** Internationally, traumatic injury causes 10% of deaths (5.8 million individuals) [1]. Highlighting those with an increased risk of mortality may focus care. The Risk-stratification of Emergency Department suspected Sepsis (REDS) score predicts mortality in patients with suspected sepsis. [2] Major traumatic injury also evokes an innate immune response [3]; therefore, the REDS score may also predict mortality in major trauma. This study aimed to evaluate whether the REDS score could predict mortality in adult major trauma patients and help risk-stratify care.

**Methods** All major trauma patients presenting to a London Major Trauma Centre in December 2023 were included. Data was collected from electronic patient records (age, sex, altered mental state, systolic blood pressure, respiratory rate, lactate, albumin, INR, refractory hypotension, and in-hospital mortality). These variables were used to calculate each patient’s REDS score. A chi-squared test was used to assess whether REDS score correlated with mortality.

**Results** There were 90 major trauma patients. 38 were excluded due to missing data. 52 patients were included in the final analysis. 61% were male, with a mean age of 58, and a 9.6% mortality (n = 5). A REDS score of 4 was associated with increased mortality. No patients with a REDS score of 3 died. 33% with a score of 4, 40% with a score of 5, and 67% with a score of 6 died. The association between REDS score and mortality was significant (5%, *p* < 0.001).

**Discussion** This novel use of the REDS score may highlight adult major trauma patients at increased risk of mortality. Further research could be undertaken, with a larger sample size, to assess which REDS score variables are most significantly associated with mortality in major trauma, to incorporate injury mechanism, and to evaluate how a modified REDS score may be used in risk-stratification and optimising major trauma patient care.

**References**

1. Injuries and Violence: the facts. World Health Organization, 2012. [Oct;2020];https://www.who.int/violence_injury_prevention/key_facts/en/.
2. Sivayoham N, Blake LA, Tharimoopantavida SE, Chughtai S, Hussain AN, Cecconi M, et al. The REDS score: a new scoring system to risk-stratify emergency department suspected sepsis: a derivation and validation study. BMJ Open [Internet]. 2019 Aug [cited 2024 Sep 18];9(8):e030922. Available from: https://bmjopen.bmj.com/content/9/8/e030922.
3. Huber-Lang M, Lambris JD, Ward PA. Innate immune responses to trauma. Nature Immunology [Internet]. 2018 Mar 5 [cited 2024 Sep 18];19(4):327–41. Available from: https://www.nature.com/articles/s41590-018-0064-8

### O2. Complications associated with pre-hospital open thoracostomy

#### Lesley Blake^1^, James Raitt^2^, Edward Cervetto Norris^2^

##### ^1^Oxford University Hospital, Oxford, UK; ^2^Thames Valley Air Ambulance, Oxford, UK

**Correspondence:** Lesley Blake (lesley.blake@nhs.net)

*Scandinavian Journal of Trauma, Resuscitation and Emergency Medicine* 2024, **32(S1)**: O2

**Background** Tension pneumothorax is a common injury [2] within major trauma. Open thoracostomy is an effective method by which to treat this life threatening problem. Open thoracostomy, however, is not a benign procedure, with complication rates estimated at 10–30% [1–3] when performed both pre-hospital and in hospital. There is little literature available to assess the incidence of long term complications in prehospital performed open thoracostomy.

**Method** A retrospective case series of all patients who underwent open thoracostomy during a 58 month period with a prehospital air ambulance service. The electronic patient notes were assessed for indications for thoracostomy, direct complications (persistent air leak, infection, damage to lung, damage to neurovascular structures), survival and death.

**Results **Within the period examined, 77 patients underwent 134 prehospital thoracotomies. 87% of these met the criteria for the procedure. Complications occurred in 12.6% of prehospital thoracostomies. 18% were persistent air leak, and a further 18% had bronchopleural fistula demonstrated on CT. Failure to decompress the chest occurred in 18%, and there was 1 incidence (6%) of vessel injury and infection respectively. Of the 77 patients studied, 52% survived to repatriation or discharge. 48% died, with 76% of these dying in the first 72 h. There were no deaths and no surgeries caused by complications from thoracostomy.

**Discussion** A 12.6% complication rate is similar to that described in the literature [1–3], with no major complications occurring within the data collection period. Whilst more patients met the criteria for insertion than previous literature has described it remains important to ensure this invasive procedure is clinically justified.

**References**

1. Leech C, Revell M, Porter K, Steyn R. The prehospital management of chest injuries: a consensus statement. Faculty of Pre-hospital Care, Royal College of Surgeons of Edinburgh. Emergency Medicine Journal [Internet]. 2007 Mar 1;24(3):220–4. Available from: https://www.ncbi.nlm.nih.gov/pmc/articles/PMC2660039/.
2. Mohrsen S, McMahon N, Corfield A, McKee S. Complications associated with pre-hospital open thoracostomies: a rapid review. Scand J Trauma Resuscit Emerg Med. 2021 Dec;29(1).
3. Bailey RC. Complications of tube thoracostomy in trauma. Emerg Med J. 2000 Mar 1;17(2):111–4.

### O3. Prevalence of hypofibrinogenemia in polytraumatized patients upon admission to a german level-1 trauma centre and its association with core outcomes

#### Clemens Grimm^1^, Hanna Illian^1^, Christopher Spering^2^, Nicolas Fink^1^, Nils Kunze-Szikszay^1^

##### ^1^Department of Anaesthesiology, University Medical Center Göttingen, Germany; ^2^Department of Trauma Surgery, Orthopedics, and Plastic Surgery, University Medical Center Goettingen, Germany

**Correspondence:** Clemens Grimm (clemens.grimm@umg.eu)

*Scandinavian Journal of Trauma, Resuscitation and Emergency Medicine* 2024, **32(S1)**: O3

**Background** Hypofibrinogenemia is an independent risk factor for mortality in trauma patients with fibrinogen known to be the first coagulation factor reaching critical low levels thus contributing to trauma-associated coagulopathy [1]. Prehospital administration of fibrinogen concentrate is feasible and improves clot stability [2]. This study aims to assess the prevalence of critically low fibrinogen levels in polytraumatized patients, identifying patients potentially benefiting from prehospital fibrinogen substitution as part of planning for an interventional trial on that issue.

**Materials and methods** This single-centre retrospective study analysed local data from the TraumaRegister DGU® including adult trauma patients (age ≥ 16, Injury Severity Score (ISS) ≥ 16) that were primarily admitted to our Level-1 Trauma Centre from 2013 to 2019. Data analysis utilised Python libraries, with missing data imputed using k-nearest neighbours and class imbalances addressed via SMOTE. Logistic regression assessed the association between fibrinogen levels and clinical outcomes, adjusting for age, ISS, and GCS.

**Results** 847 polytraumatized patients were included, with 5.9% (n = 50) presenting with fibrinogen levels ≤ 150 mg/dL, 17.6% (n = 149) with levels between 150 and 200 mg/dL, and 76.5% (n = 648) with levels > 200 mg/dL. Logistic regression analysis, adjusting for fibrinogen levels, age, ISS and GCS, revealed that patients with fibrinogen levels ≤ 150 mg/dL had substantially higher odds of requiring RBC transfusion within the first 48 h of ICU admission (OR: 1.69, 95% CI 1.47–1.93, p < 0.001), as did those with fibrinogen levels between 150 and 200 mg/dL (OR: 1.56, 95% CI 1.39–1.75, *p* < 0.001). Fibrinogen levels ≤ 150 mg/dL were independently associated with increased mortality (OR: 1.38, 95% CI 1.15–1.66, p < 0.001) compared to those with fibrinogen levels > 200 mg/dL. Fibrinogen levels between 150 and 200 mg/dL did not significantly predict higher mortality (OR: 1.08, 95% CI 0.92–1.28, *p* = 0.354).

**Conclusions** A substantial proportion of polytraumatized patients was admitted with hypofibrinogenemia. Fibrinogen levels ≤ 150 mg/dL were significantly associated with poorer outcomes. Our results underline the potential benefits of prehospital fibrinogen substitution and support the need for further clinical trials to evaluate its benefits in bleeding trauma patients.

**References**

1. McQuilten ZK, Wood EM, Bailey M, Cameron PA, Cooper DJ. Fibrinogen is an independent predictor of mortality in major trauma patients: a five-year statewide cohort study. Injury. 2017 May;48(5):1074–81.
2. Ziegler B, Bachler M, Haberfellner H, Niederwanger C, Innerhofer P, Hell T, et al. Efficacy of prehospital administration of fibrinogen concentrate in trauma patients bleeding or presumed to bleed (FIinTIC): A multicentre, double-blind, placebo-controlled, randomised pilot study. Eur J Anaesthesiol EJA. 2021 Apr;38(4):348.

### O4. Inverted sling position for extraction from water (I-SPEW)

#### Espen Fevang^1^, Sveinung Tjessheim^1^

##### ^1^Department of Anaesthesiology and Intensive Care, Stavanger University Hospital, Stavanger, Norway, No. 330 Squadron Royal Norwegian Air Force

**Correspondence:** Espen Fevang (esfevang@gmail.com)

*Scandinavian Journal of Trauma, Resuscitation and Emergency Medicine* 2024, **32(S1)**: O4

**Background** Drowning continues to be a serious cause of morbidity and mortality worldwide. Unconscious drowning victims without life jackets are usually found submerged face down, indicating a filled airway [1]. When located by a helicopter with a hoisting system the patient is normally extricated in a partially upright or supine position and is placed supine for CPR when entering the helicopter. A major complication concerning airway management and resuscitation after drowning is blockage of the airway due to water and regurgitation matter as it interferes with ventilation and delays endotracheal intubation [2]. Commencing draining of the airway during the hoisting procedure may save time before establishing a secure airway, which is associated with improved patient outcomes [3]. We propose an alternative hoisting procedure, extracting the patient in an inverted position, to facilitate drainage of the airway before resuscitation is commenced. If our method can be considered safe, reliable and does not delay the hoisting operation, it may warrant a full-scale RCT investigating airway management after drowning.

**Method** We tested the inverted hoisting procedure on dummies and people in different scenarios, deliberately inducing spin, pendulums and known complications during hoisting operations. After standardizing the method, several hoistings in each position were performed in a randomized order and timed.

**Results** Mean time to extraction from water was 14.75 s in the inverted position, compared to 21.7 s in the traditional position, creating a non-significant mean difference of 6.75 s (*p* = 0.137) in favour of the inverted position. No adverse events occurred during hoisting in either position.

**Conclusion** In our pilot project, the alternative hoisting procedure did not delay the procedure, and no adverse events occurred. Based on these results, we aim to conduct an RCT investigating whether airway management after drowning can be improved using an inverted hoisting method.

**Acknowledgements**

No. 330 Squadron Royal Norwegian Air Force.

**References**

1. Caruso JL. Decomposition changes in bodies recovered from water. Acad Forensic Pathol. 2016 Mar;6(1):19–27.
2. Szpilman D, Bierens JJ, Handley AJ, Orlowski JP. Drowning. N Engl J Med. 2012;366(22):2102–10.
3. Yoshimura S, Kiguchi T, Nishioka N, Ikeda N, Takegawa M, Miyamae N, Sumida Y, Kitamura T, Iwami T. Association of pre-hospital tracheal intubation with outcomes after out-of-hospital cardiac arrest by drowning comparing to supraglottic airway device: a nationwide propensity score-matched cohort study. Resuscitation. 2024 Apr;197:110,129.

### O5. NHRAF out of hospital cardiac arrest benchmarking project

#### Kirsten Raphael^1^, Hannah Trebilcock^2^, Nigel Lang^2^, James Raitt^3^, Adam Pitcairn^4^, Tom Maxwell^5^, Sarah Thatcher^5^, Neil MacKay^6^, Patrick Duncan^7^, Tom Waters^8^, Chris Gough^9^, Matthew Stringfellow^10^, Kate Lachowycz^11^

##### ^1^Barts Health Trust, London, UK; ^2^Devon Air Ambulance, Exeter, UK; ^3^Thames Valley Air Ambulance, Stokenchurch, UK; ^4^Essex & Herts Air Ambulance, Colchester, UK; ^5^Hampshire and Isle of Wight Air Ambulance, Southampton, UK; ^6^North West Air Ambulance, Knowsley, UK; ^7^Great North Air Ambulance Service, Stockton-on-Tees, UK; ^8^Midlands Air Ambulance, Shifnal, UK; ^9^Lincs & Notts Air Ambulance, Lincoln, UK; ^10^The Air Ambulance Service, Rugby, UK; ^11^East Anglian Air Ambulance, Norwich, UK

**Correspondence:** Kirsten Raphael (kirsten.raphael1@nhs.net)

*Scandinavian Journal of Trauma, Resuscitation and Emergency Medicine* 2024, **32(S1)**: O5

**Background **The National HEMS Research and Audit Forum (NHRAF) was founded in 2020 to establish an inclusive forum within UK HEMS and land-based Pre-Hospital Critical Care Teams (PHCCT) with the aim of improving patient experience through collaborative research and audit. One objective was to host collective data and share aggregated results. This benchmarking project developed performance indicators for medical Out of Hospital Cardiac Arrest (OOHCA).

**Method** A working group was established who selected performance indicators that were meaningful and feasible to collect as markers of management of OOHCA. Metrics selected were:

- Median time 999 call to mobilization (dispatch time)
- Patients conveyed to hospital (%)
- ROSC at hospital rate:
  - ROSC at hospital/total number of arrests (%)
  - ROSC at hospital/total number of patients conveyed to hospital (%)
- Combined first and second pass success rate for endotracheal intubation (%)
- Survival to discharge (%)

Each service was able to identify their own results against anonymized results of the other participating services and the means and medians.

**Results** 10 services participated. The median dispatch time was 9 min (range 2–14), median rate of ROSC at hospital was 82% (range 40–100%) and median percentage of arrests where intubation was attempted 36% (range 6–61%). All institutions had a success rate of > 85% for intubation within 2 attempts (intubation success within 2 attempts range 86–100%).

**Discussion** This benchmarking program has allowed individual institutions to reflect on their practice and processes. The greatest variation in results was seen in median time to dispatch; these results will allow services to discuss and compare their dispatch methods. This is the first collaborative benchmarking exercise of its type in UK pre-hospital critical care and allows a unique opportunity for participating services to refine and develop their processes. After an initial period, the benchmarking will be opened to other PHCCTs.

**Acknowledgements**

Special thanks go to the National HEMS Research and Audit Forum for having facilitated bringing the participating organizations together and making this project possible.

### O6. Redicting severe injuries using a machine learning algorithm trained on emergency department triage notes: is this the future of trauma team activation?

#### Oscar Lapidus^1^, Salvar Jóhannsson^1^, Martin Jacobsson^2^, Denise Bäckström^3^, Folke Hammarqvist^1^, Andreas Wladis^3^, Rebecka Rubenson Wahlin^1^

##### ^1^Department of Clinical Science, Intervention and Technology, Karolinska Institutet, Stockholm, Sweden; ^2^Department of Biomedical Engineering and Health Systems, KTH Royal Institute of Technology, Huddinge, Sweden; ^3^Department of Biomedical and Clinical Sciences, Linköping University, Linköping, Sweden

**Correspondence:** Oscar Lapidus (oscar.lapidus@ki.se)

*Scandinavian Journal of Trauma, Resuscitation and Emergency Medicine* 2024, **32(S1)**: O6

**Background** Trauma team activation is typically prompted by obtaining prehospital information and evaluating whether any predefined TTA criteria are fulfilled. However, the performance of such criteria-based systems may be limited by their relative simplicity. No previous studies have examined the possibility of using artificial intelligence to predict the risk of severe injuries and evaluate the need for TTA.

**Method **3317 trauma patients treated at Södersjukhuset were used to train a local instance of AI Sweden’s large language model GPT-Sw3 356 M modified for binary classification. The predictor variable was emergency department triage notes and the binary outcome was NISS ≥ 16. The triage notes were in the form of semi-structured free text written either by the ambulance personnel or triage nurse in the emergency department, typically one to three sentences regarding the primary complaint, mechanism of injury and brief patient history. The model was then tasked to estimate the probability of NISS ≥ 16 in a dataset of 585 previously unseen patients. Model performance was assessed by evaluating the receiver operating characteristics curve as well as by calculating the classification sensitivity and specificity at the point with the maximum F_1_ score.

**Results **The LLM had a sensitivity of 78% and specificity of 49% in predicting which patients would have NISS ≥ 16 based on emergency department triage notes (AUROC = 0.673). For comparison, the level 1 TTA criteria had a sensitivity of 27% in the same cohort, albeit with a higher specificity of 80%.

**Conclusion** The machine learning model’s performance in classification was fair, with a more balanced ratio of sensitivity to specificity compared to the TTA criteria, and impressive given the limited information available from the triage notes. Further investigation of the feasibility of using machine learning for trauma triage and its performance in relation to current TTA guidelines is warranted.

### O7. Pre-hospital predictors of brainstem herniation in patients with severe traumatic brain injury: a retrospective analysis

#### Adam J. R. Watson^1^, Julian Hannah^2^, Peter Owen^3^, James O. M. Plumb^4^

##### ^1^Clinical & Experimental Sciences, Faculty of Medicine, University of Southampton, Southampton, UK; ^2^University Hospital Southampton NHS Foundation Trust, Southampton, UK; ^3^Hampshire & Isle of Wight Air Ambulance, Southampton, UK; ^4^Perioperative & Critical Care Theme, NIHR Southampton Biomedical Research Centre, Southampton, UK

**Correspondence:** Adam J. R. Watson (adam.watson@doctors.org.uk)

*Scandinavian Journal of Trauma, Resuscitation and Emergency Medicine* 2024, **32(S1)**: O7

**Background **The devastating consequences of traumatic brain injury (TBI) can include brain herniation secondary to increased intracranial pressure (ICP). In the pre-hospital setting, clinical signs such as fixed dilated pupils (FDPs) or Cushing’s reflex are reported to be poor predictors of raised ICP [1]. We aimed to assess the ability of pre-hospital clinical signs to predict impending herniation.

**Methods **We included consecutive ≥ 16 years olds with blunt TBIs who received Pre-Hospital Emergency Anaesthesia (PHEA) from Hampshire & Isle of Wight Air Ambulance. Pre-hospital data collected included Glasgow Coma Score (GCS), Heart Rate (HR), Systolic Blood Pressure (SBP), and pupillary light reflexes. The first Computerised Tomography (CT) head scans were reviewed for evidence of brainstem herniation. We tested the predictive ability of each clinical sign at various meaningful thresholds, before combing those that performed best to produce a composite score. Area Under Receiver Operating Characteristic curve (AUC) and sensitivity/specificity data is reported. The study used routinelly collected data and was approved as a service evaluation.

**Results** We included 104 patients (mean age 56.4 years, 66% male). On HIOWAA arrival, median GCS was 6 (IQR 4–9) and 36 patients (35%) had unilateral or bilateral FDP(s). On hospital arrival, 19 patients (18%) had radiological evidence of brainstem herniation on hospital arrival. In our model, 1 point is awarded for best GCS ≤ 5, any HR ≤ 70, any SBP ≥ 180 mmHg, or any FDP(s), and this score has good predictive performance (AUC 0.867). If a threshold of ≥ 2 is adopted to predict herniation, the sensitivity is 0.95 (95% CI 0.74–1.00) and specificity is 0.64 (95% CI 0.52–0.74).

**Conclusion** Our findings suggests that accurate pre-hospital prediction of impending brainstem herniation may be possible, but requires validation in larger prospective studies.

**Reference**

1. Ter Avest E, Taylor S, Wilson M, Lyon RL. Prehospital clinical signs are a poor predictor of raised intracranial pressure following traumatic brain injury. Emerg Med J 2021;38:21–6. 10.1136/emermed-2020-209635.

### O8. A Shocking use of maps

#### Emma Moore^1^, Christopher Arrowsmith^1^, Jans Gerhards^2^

##### ^1^University Hospitals Bristol NHS Trust, Bristol, UK; ^2^Great Western Air Ambulance Charity GWAAC, Bristol, UK

**Correspondence:** Christopher Arrowsmith (Christopher.arrowsmith@uhbw.nhs.uk)

*Scandinavian Journal of Trauma, Resuscitation and Emergency Medicine* 2024, **32(S1)**: O8

**Background** Across the UK, survival from out of hospital cardiac arrest (OHCA) remains low at c.8% [1]. Accessing a defibrillator within 3–5 min of cardiac arrest can increase the chances of survival by 40% [2]. Background health factors and population density contribute to higher rates of OHCA in less affluent areas, therefore areas where public access defibrillators (PADs) would be effective. Unfortunately, current evidence suggests that PADs are disproportionately placed in more affluent areas and that there is a discrepancy between OHCA occurrence and PAD density [3]. In our region we do not know if the current PAD placement is equitable from a socio-economic perspective, and it is not clear where placing new PADs would be most effective.

**Method** Using data from South West Ambulance Service and UK Government Websites we have developed an interactive ‘heat-map’ covering 10,000 km^2^ where incidents requiring defibrillators are overlayed with socioeconomic factors, population densities and PADs. We have used a mathematical distance improvement system to show where placement of a PAD can most effectively reduce time to defibration from the initial 999 call.

**Results** Results are striking, in one area over the past 12 months there have been 117 incidents and only 1 existing PAD, whilst in another there have been only 13 incidents and 4 existing PADs. There is an inverse correlation between PAD placement and areas of multiple depravation. Using the distance improvement overlay we can identify where placement of a PADs can significantly reduce time to defibrillation.

**Conclusion** This map is a potent tool. It confirms our hypothesis of a lack of equity with PAD placement, and importantly can direct new PAD placement to improve this.

**Discussion** We will engage with the local councils across our region in targeting the placement of new PADs to provide care to the most vulnerable people.

**References**

1. Perkins G. Out of Hospital Cardiac Arrest Outcomes (OHCAO) Database. Funded by Resuscitation Council. https://warwick.ac.uk/fac/sci/med/research/ctu/trials/ohcao/ accessed on 17/09/24.
2. Joint Parliamentary Briefing: Public Access to Defibrillators 2023 https://www.resus.org.uk/sites/default/files/2023-07/Public%20Access%20to%20Defibrillators%20-%20Joint%20Briefing.pdf accessed on 17/09/24.
3. Brown T, Perkins G, Smith C et al. Are there disparities in the location of automated external defibrillators in England? *Resuscitation.* 2022; 170, 28–35.

### O9. The pathophysiology of shock following traumatic arterial injuries and the implications for resuscitation. Part 2: an observational study of injured patients

#### Carl Evans^1^, Robbie Lendrum^2^, Ewoud ter Avest^2^, Zane Perkins^2^

##### ^1^East Sussex Healthcare NHS Trust; ^2^London’s Air Ambulance

**Correspondence:** Carl Evans (carl.evans@nhs.net)

*Scandinavian Journal of Trauma, Resuscitation and Emergency Medicine* 2024, **32(S1)**: O9

**Introduction and Aims** We hypothesise, following a review of the literature [1], that an injury to an elastic artery will result in failure of the arterial pressure reservoir, resulting in a low diastolic pressure and thus an impairment of coronary perfusion. This suggests that the nature of arterial injury extends beyond that of volume loss, where damage to the arterial vasculature directly impairs coronary perfusion thus inhibiting cardiac compensation following injury, leading to rapid, profound shock and subsequent cardiac arrest. We sought to identify this physiological pattern in injured patients.

**Methods** An observational study utilising haemodynamic, biochemical and traumatic cardiac arrest data in patients with and without arterial injury was conducted to identify preliminary data to support the hypothesis that arterial injury impairs the arterial pressure reservoir, impairing the diastolic blood pressure and thus coronary perfusion.

**Results** Haemodynamic data from ten cases illustrates how arterial injury can lead to a wide pulse pressure and a low diastolic pressure (< 50% of the systolic). We suggest that a low diastolic pressure leads to a secondary cardiac injury as the myocardium is dependent upon diastolic pressure (particularly within the left ventricle) to maintain coronary perfusion. To demonstrate this, in a cohort of 135 consecutive trauma patients, we identified statistically significant (*p* < 0.001) levels of Heart-Type Fatty Acid Binding Protein (a marker of myocardial ischaemia) in patients following arterial injury compared to shocked and non-shocked controls.

**Hypothesis/Conclusion** Our preliminary data supports the hypothesis that the nature of arterial injury extends beyond that of volume loss, where damage to the arterial vasculature prevents the arterial pressure reservoir from functioning, directly impairing coronary perfusion therefore inhibiting cardiovascular compensation following injury leading to rapid, profound shock and subsequent cardiac arrest. Further research is planned to further develop and strengthen this association.

**Reference**

1. Davies JE, Hadjiloizou N, Leibovich D, Malaweera A, Alastruey-Arimon J, Whinnett ZI, et al. Importance of the aortic reservoir in determining the shape of the arterial pressure waveform: the forgotten lessons of Frank. Artery Res [Internet]. 2007;1(2):40–5. Available from: https://www.sciencedirect.com/science/article/pii/S187293120700155X.

## Poster Presentations

### P1. Diagnostic Accuracy of Clinical Examination for Identification of Life-Threatening Torsos Injuries: a Meta-Analysis

#### Thomas Durrands^1^, Mark Murphy^2^, Jared M Wohlgemut^1^, Henry D De’Ath^1^, Zane B Perkins^1^

##### ^1^Centre for Trauma Sciences, Blizard Institute, Queen Mary University of London; ^2^Frimley Health NHS Foundation Trust

**Correspondence:** Thomas Durrands (thomas.durrands@nhs.net)

*Scandinavian Journal of Trauma, Resuscitation and Emergency Medicine* 2024, **32(S1)**: P1

**Background** Timely identification and management of life-threatening torso injuries is a fundamental objective of the initial assessment of the trauma patient. The accuracy of physical examination in identifying these injuries is unclear. The objective of this meta-analysis was to determine the diagnostic accuracy of physical examination during the primary survey of adult torso trauma patients [1].

**Methods** The study adhered to the Cochrane Handbook for Systematic Reviews of Diagnostic Test Accuracy [2]. A systematic literature search (Medline, Embase, Cochrane Central) was performed independently by two authors to identify original studies that reported the accuracy of torso physical examination in injured adult patients. The Quadas-2 tool was used for the risk of bias assessment [3]. A random-effects model was used to conduct the meta-analysis.

**Results** From 4388 citations identified, thirty-five studies (54,243 patients) were included in the meta-analysis. The pooled accuracy of physical examination was: chest (sensitivity 76% [95% confidence interval 65–85%]; specificity 85% [76–91%]), abdomen (sensitivity 64% [48–78%]; specificity 75% [60–86%]), and pelvis (sensitivity 78% [67–86%]; specificity 90% [84–94%]). Sensitivity of physical examination was more accurate when GCS was ≥ 13 compared with < 13 or not specified (90% [84–93%] versus 63% [56–68%]) and within a hospital setting compared with pre-hospital (76% [68–84%] versus 46% [36–56%]). No statistical difference was identified in sensitivity in penetrating (80% [70–87%]) versus blunt trauma (68% [61–74%]). Limitations included heterogeneity of index criteria and reference standards, and medium-to-high risk of bias of included studies.

**Conclusions** The ability of physical examination to accurately identify life-threatening torso injuries is limited due to high rates of false positives and negatives, particularly in abdominal trauma. Reduced consciousness and the pre-hospital setting worsen accuracy. The relative inaccuracy of physical examination should be taken into consideration during clinical decision-making, and where possible, clinical or radiological adjuncts should be used to support the assessment of torso trauma.

**References**

1. Thomas Harry Durrands, Mark Murphy, Jared M Wohlgemut, Henry D De’Ath, Zane B Perkins, Diagnostic accuracy of clinical examination for identification of life-threatening torsos injuries: a meta-analysis, British Journal of Surgery, Volume 110, Issue 12, December 2023, Pages 1885–1886, 10.1093/bjs/znad285
2. Cochrane. Cochrane handbook for systematic reviews of diagnostic test accuracy.
3. Whiting PF, Rutjes AW, Westwood ME, Mallett S, Deeks JJ, Reitsma JB, et al. QUADAS-2:a revised tool for the quality assessment of diagnostic accuracy studies. Ann Intern Med.2011;155(8):529–36.

### P2. Transfer and handover of trauma patients in resus and its effect on patient outcomes

#### Katherine Lund^1^

##### ^1^Major Trauma Centre, Queen’s Medical Centre, Nottingham, UK

**Correspondence:** Katherine Lund (Katherine.Lund@ULH.nhs.uk)

*Scandinavian Journal of Trauma, Resuscitation and Emergency Medicine* 2024, **32(S1)**: P2

**Background** A patient arrives in resus; What happens next? Transfer to bed or handover? The IMIST-AMBO^#^ protocol, as demonstrated in a report by NSW Ambulance Service [1], states that stable patients should be transferred after a ‘hands-off’ handover, with unstable patients transferred before handover to ensure time critical treatments are started as soon as possible. This audit aims to establish whether some more consistency with these practices would be beneficial. It also looks at the effect this has on issues such as noise, distractions and communication [2].

**Methods** This audit included trauma patients presenting by ambulance to QMC resus over a 10-day period. The following was assessed for each patient: stability (stable/unstable), time of transfer (before/during/after handover), length of handover (seconds), time to primary survey (minutes), and the number and types of issues raised. The term ‘appropriate transfer’ refers to either the transfer of a stable patient after handover, or an unstable patient before handover. Associations between each outcome were also reviewed.

**Results** 35% of transfers were appropriate (22% in reaudit). When the length of handover was < 90 s, or when time to primary survey was < 4 min, or when ≤ 1 issues were raised, 50% of these transfers were appropriate (0%, 0% and 29% respectively in reaudit). The average number of issues was 1.2 (0.9 in reaudit), the most common being noise (lack of capacity in reaudit).

**Conclusions** There is no clear guidance on which patients should be transferred before or after handover. There is no positive association between the time of transfer and either the length of handover, time to primary survey or number of issues raised. There was an improvement in the number of issues raised in the reaudit. This audit has raised awareness of the need for a clear protocol regarding the transfer of patients in resus.

**References**

1. Iedema, R. and Ball, C. (2010) NSW Ambulance/ Emergency Department Handover Project Report. Sydney: NSW Health & UTS Centre for Health Communication.
2. Dawson S, King L, Grantham H. (2013) Review article: Improving the hospital clinical handover between paramedics and emergency department staff in the deteriorating patient. *Emergency Medicine Australasia,* 25(5), pg. 393–405. 10.1111/1742-6723.12120.

^#^IMIST-AMBO stands for Identification, Mechanism/Medical complaint, Injuries, Signs, Treatments; Allergies, Medications, Background history, Other information.

### P3. Designing a human-centred, AI equipped, digital decision support interface to expedite the management of major trauma

#### Sean Buchanan^1^, Zane Perkins^1^

##### ^1^Centre for Trauma Sciences, Blizard Institute, Queen Mary University of London

**Correspondence:** Sean Buchanan (Sean.buchanan@nhs.net)

*Scandinavian Journal of Trauma, Resuscitation and Emergency Medicine* 2024, **32(S1)**: P3

**Background** Traumatic injuries are a leading cause of morbidity and mortality globally. Specialist trauma centers have demonstrated better outcomes for polytrauma patients. A timely and accurate identification of trauma-induced coagulopathy (TIC) could enhance pre-hospital interventions and streamline surgical decision-making between damage control and definitive surgery. However, the current major trauma alert system may not provide adequate information to estimate TIC risk. This study aimed to document clinical workflows and decision points to guide the development of a clinical decision support system with AI integration, potentially reducing treatment delays.

**Materials and methods** This mixed-methods study involved direct observation of the London Air Ambulance (LAA), London Ambulance Service (LAS), and trauma teams at King's College Hospital and the Royal London Hospital. The Systems Engineering for Patient Safety (SEIPS) framework was applied to analyse the socio-technical environment in which the new alert system would operate. An audit of retrospective case reviews was conducted to assess the frequency and availability of key trauma variables communicated before patient arrival. Workshops with major trauma consultants, surgeons, and intensivists were held to guide user-driven interface design, resulting in high-level user stories for the technical framework.

**Results** The current alert system inconsistently communicated vital information. Mechanism of injury (93.8%) and head injury presence (86.6%) were commonly reported, while critical factors like vital signs, ultrasound findings, and fluid resuscitation volume were less frequently communicated (< 50%). SEIPS analysis revealed barriers such as the physical limitations of using electronic devices in unpredictable environments and concerns about AI predictions affecting professional autonomy.

**Conclusions** The current system inadequately relays key clinical information needed to expedite trauma care. An AI-enhanced alert system could support decision-making and improve outcomes, though challenges surrounding trust and user interface design must be addressed.

### P4. The use of light on skid in Norwegian helicopter emergency service, a retrospective study

#### Thomas Nordgaard Dahle^1^, Anders Myrvoll-Nordgaard^3^, William Ottestad^1,2^, and Martin Samdal^4^.

##### ^1^Department of Research, Air Ambulance Foundation, Oslo, Norway; ^2^Air Ambulance Department, Oslo University Hospital, Oslo, Norway; ^3^Norwegian Air Ambulance Helicopter, Oslo, Norway; ^4^Air Ambulance Department, Vestre Viken Hospital, Drammen, Norway

**Correspondence:** Thomas Nordgaard Dahle (thomas.nordgaard.dahle@norskluftambulanse.no)

*Scandinavian Journal of Trauma, Resuscitation and Emergency Medicine* 2024, **32(S1)**: P4

**Background** The Norwegian helicopter emergency medical services (HEMS) are equipped to carry out rescue operations. In challenging terrain or confined spaces where nearby landing sites are unavailable, hovering light on skid can provide a practical solution for swiftly accessing patients. The helicopter medical emergency service in Norway consist of 13 bases and 99% of the population can be reached within 35 min flight time [1]. The Norwegian Air Ambulance Helicopter operate a fleet of helicopters from Airbus with the H135 and H145, and from Leonardo with the AW 139 [2]. Our study aimed to examine epidemiological factors associated with light on skid operations conducted by Norwegian helicopter emergency service.

**Method** We reviewed patient descriptors; including age, sex, injury severity and diagnosis during a five-year period from 2018 to 2022. Data are presented as medians with quartiles, except for National Advisory Committee for Aeronautics (NACA) scores, which are presented as modes.

**Result** During the study period, 460 patients were rescued using light on skid operations. Of these, 165 (53%) were men and 145 (47%) were women. The median age was 46 years (27–59). The most common NACA score was 3, with 53 patients (17%) having a score above 4, indicating a potential or actual life-threatening condition. Trauma-related injuries were present in 242 (78%) patients, while 72 (22%) had medical conditions.

**Conclusion** A considerable proportion of patients rescued by the light on skid procedure had a NACA score ≥ 4 underpinning the importance of rapid rescue in the Norwegian helicopter emergency service.

**References**

1. Jagtenberg CJ et al. "Utopia for Norwegian helicopter emergency medical services: Estimating the number of bases needed to radically bring down response times, and lives needed to be saved for cost effectiveness." PLOS ONE. 2023; 18(3): e0281706.
2. Luftambulansetjenesten HF "Basene våre." Retrieved 11 september 2024, 2022 ; from https://www.luftambulanse.no/basene-vare.

### P5. Technological advances necessitating a move away from a watchful waiting management strategy in digital necrosis

#### Rishabh Jain^1^, Andrew R McKean^2^, Shakeel M Rahman^2^, Asmat H Din^2^, Edmund Fitzgerald O’Connor^2^

##### ^1^Department of Trauma and Orthopaedic Surgery, Guys and St Thomas’ NHS Foundation Trust, London, UK; ^2^Department of Plastic Surgery, Guys and St Thomas’ NHS Foundation Trust, London, UK 

**Correspondence:** Rishabh Jain(rishmiester@gmail.com)

*Scandinavian Journal of Trauma, Resuscitation and Emergency Medicine* 2024, **32(S1)**: P5

**Background** In the context of major trauma and cardiac arrest, high dose inotropic and vasopressor usage can predispose to distal digital necrosis [1]. Digital necrosis management traditionally involves a watchful waiting approach, allowing demarcation between viable and necrotic tissue to maximize tissue preservation [2]. However, the increasing dependence on touch screen technology has introduced challenges for patients with necrotic digits, as capacitive screens require conductive tissue, which necrotic tissue lacks [3]. This paper explores the impact of smartphone use on the management of digital necrosis, proposing alternative strategies that could better support patient functionality.

**Method** A comprehensive literature review was conducted, examining cases of digital ischemia and necrosis in relation to modern touchscreen technology. Specific attention was given to how capacitive touchscreens, which rely on electrical conductivity, are incompatible with necrotic or dry tissue. Additionally, alternative management approaches, such as earlier terminalization or the use of conductive gloves, were considered to address the functional needs of patients reliant on smartphones.

**Results** The literature review identified that patients with necrotic fingers face significant challenges in using smartphones due to the non-conductive nature of necrotic tissue. While the traditional expectant management strategy remains clinically sound for tissue preservation, it may prolong functional impairment [2]. Alternative approaches, including early terminalization, allow patients to regain smartphone use and social connectivity earlier. Conductive gloves also offer an interim solution while following a watchful waiting approach.

**Conclusion** With the widespread reliance on smartphones, the traditional watchful waiting management of digital necrosis may no longer fully meet patients' needs for functional independence. Early terminalization or the use of conductive gloves offers a way to improve quality of life by restoring the ability to interact with touchscreens sooner. A shift in the management paradigm, considering these technological and functional needs, may be necessary.

**References**

1. Landry GJ, Mostul CJ, Ahn DS, McLafferty BJ, Liem TK, Mitchell EL, et al. Causes and outcomes of finger ischemia in hospitalized patients in the intensive care unit. J Vasc Surg. 2018;68(5):1499–1504.
2. Thibaudeau S, Serebrakian AT, Gerety PA, Levin LS. An Algorithmic Approach to the Surgical Treatment of Chronic Ischemia of the Hand: A Systematic Review of the Literature. Plast Reconstr Surg. 2016;137(5):818e–828e.
3. Melbourne Hand Rehab. Touchscreen difficulties? Do you have Zombie Finger? [Internet]. 2019. Accessed: 12th April 2021. Available at: Touchscreen Difficulties? Do you have Zombie Finger? [https://melbournehand.com.au/touchscreen-difficulties-do-you-have-zombie-finger/].

### P6. Splenic function following angioembolisation for blunt splenic injury: a systematic review

#### William J Broadhurst^1^, Timothy S Gomm^1^, Henry D De’Ath^2^

##### ^1^Department of General Surgery, University College London Hospital, London, UK; ^2^Centre for Trauma Sciences, Blizard Institute, Queen Mary University of London, London, UK

**Correspondence:** William J Broadhurst (william.broadhurst2@nhs.net)

*Scandinavian Journal of Trauma, Resuscitation and Emergency Medicine* 2024, **32(S1)**: P6

**Background** Splenic artery angioembolisation (SAE) plays an important role in the nonoperative management of blunt splenic injury. In contrast to splenectomy, there is little guidance concerning immune function, and consequently the need for antimicrobial prophylaxis, following SAE. A systematic review was conducted to evaluate the current literature on the topic.

**Method** A systematic review of the currently available literature was performed using the MEDLINE database. Original articles were eligible if they reported at least one marker of splenic function following SAE. Animal studies, literature reviews and case reports including only a single patient were excluded. Two researchers independently assessed the eligibility and quality of the articles and performed the data extraction. These studies were then qualitatively analysed.

**Results** Eleven studies were included. A total of 407 patients, of which 243 had undergone SAE, 80 who had undergone splenectomy and 84 healthy controls, were included. All studies used different parameters for assessing splenic function. None reported increased rates of infection or overwhelming postsplenectomy infection following SAE. There was no statistically significant difference between the markers of immunocompetence measured in the SAE group when compared to healthy controls. Ten of the studies concluded that, by the parameters they measured, splenic function was preserved after the procedure.

**Conclusion** All but one of the included studies reported preserved splenic function following SAE. Whilst there is no single parameter that unequivocally demonstrates this, the best available evidence supports this. This may help inform clinical guidelines for post-SAE vaccination and prophylactic antibiotic practice; namely, that there is no indication to do so.

### P7. A systematic review of compression depth in infant CPR: two-finger vs. two-thumb techniques

#### David Gray^1^, Lisa Ramage^2^

##### ^1^University of Cambridge, Cambridge, UK, ^2^Cambridge Pre‑Hospital Care Programme, Cambridge, UK

**Correspondence:** David Gray (dg596@cam.ac.uk)

*Scandinavian Journal of Trauma, Resuscitation and Emergency Medicine* 2024, **32(S1)**: P7

**Background** In the US, over 15,000 children suffer a cardiac arrest annually [1], of which 3.3% of infants survive to hospital discharge [2]. According to the UK Resuscitation Council (UKRC), chest compression quality is the most important factor in improving the rate of infant survival with no neurological deficit [3]. One of the most widely reported components of chest compressions is compression depth (CCD). This systematic review compares the compression depth achieved by two different infant CPR techniques endorsed by the UKRC, the two-finger (TF) and two-thumb (TT) technique, to see if there are any differences in CCD elicited.

**Method** A systematic search of PubMed was conducted for studies published between 2014 and 2024. Randomized controlled trials (RCTs) involving infant CPR on mannequins were included. Eligible studies focused on comparing compression depth between the two-finger and two-thumb techniques. The Data was extracted and reviewed for analysis.

**Results** 11 studies, involving 714 participants, met the inclusion criteria. The data revealed that the two-thumb technique consistently achieved significantly deeper compressions than the two-finger technique across all studies (p < 0.05). This finding was consistent across different professional backgrounds, experience levels, and ventilation parameters.

**Conclusion** The two-thumb technique produces deeper chest compressions than the two-finger technique in infant CPR. Given these findings, the two-thumb approach should be considered the preferred method for delivering high-quality compressions. However, other factors, such as chest recoil, compression fraction, and rescuer fatigue, also influence overall CPR quality and warrant further investigation. Additional research is needed to fully assess the relative benefits of both techniques in infant resuscitation.

**References**

1. Holmberg MJ, Ross CE, Fitzmaurice GM, Chan PS, Duval-Arnould J, Grossestreuer AV, et al. Annual Incidence of Adult and Pediatric In-Hospital Cardiac Arrest in the United States. Circulation Cardiovascular quality and outcomes [Internet]. 2019 [cited 2024 Sep 20];12(7):e005580. Available from: https://www.ncbi.nlm.nih.gov/pmc/articles/PMC6758564/.
2. Atkins DL, Everson-Stewart S, Sears GK, Daya M, Osmond MH, Warden CR, et al. Epidemiology and outcomes from out-of-hospital cardiac arrest in children. circulation [Internet]. 2009 Mar 24 [cited 2024 Sep 20];119(11):1484–91. Available from: https://www.ahajournals.org/doi/full/10.1161/CIRCULATIONAHA.108.802678.
3. Alexandros Douvanas, Koulouglioti C, Kalafati M. A comparison between the two methods of chest compression in infant and neonatal resuscitation. A review according to 2010 CPR guidelines. The Journal of Maternal–Fetal & Neonatal Medicine [Internet]. 2017 Mar 5 [cited 2024 Sep 20];31(6):805–16. Available from: https://pubmed.ncbi.nlm.nih.gov/28282762/.

### P8. Embedding psychological services within a paediatric Major Trauma Centre (MTC): learning from the first four months of implementation, May–September 2024

#### Joshua P Roberts^1^, Jemima O’Kelly^1^

##### ^1^Major Trauma Centre, King’s College Hospital, London, United Kingdom

**Correspondence:** Joshua P Roberts (Joshua.Roberts12@nhs.net)

*Scandinavian Journal of Trauma, Resuscitation and Emergency Medicine* 2024, **32(S1)**: P8

**Background** Children and families admitted to Intensive Care Units are at risk of experiencing psychological difficulties, including post-traumatic stress [1]. Less is known regarding the prevalence of psychological distress following treatment within a paediatric major trauma pathway. MTC patients experience a traumatic mechanism of injury and potentially life altering or threatening conditions, however acuity can vary. Early studies suggest psychological provision is beneficial for patients, families and staff impacted by major trauma [2]. As a newly established paediatric psychology service within a London MTC, we aim to explore early prevalence data and feedback to help guide future development and research.

**Methods** The service model is outlined and descriptive statistics are presented from paediatric MTC patients who accessed psychological services. Ongoing feedback mechanisms from patients, families and staff are reviewed to generate themes.

**Results** A total of 48 (33 male, 15 female) paediatric (*M = *11.5 years) patients were eligible for psychological screening between May and September 2024. Of this, 43 (90%) were approached and engaged in assessment, 14 received extended therapeutic input as an inpatient. In addition, 17 family members accessed psychological support. Common mechanisms of injury: road accident (60%), serious fall (21%), stabbing (15%). The most common focus of intervention was; managing psychological trauma, hospital environment and anxiety. Feedback indicates that psychoeducation, staff support on trauma-informed care and coping in hospital represent patient and staff priorities. Themes around adultification [3] arose, notably paediatric patients on adult wards injured through community violence. Of the feedback received from this patient group (*n* = 4, 16-18 years), 100% stated it was helpful talking to a psychologist and provided an opportunity for engagement.

**Conclusions** This data helps direct psychological intervention and research within paediatric major trauma. Trauma-focused intervention, anxiety, hospital environment, staff training and engaging hard to reach communities are important areas of focus.

**References**

1. Colville G, Pierce C. Patterns of post-traumatic stress symptoms in families after paediatric intensive care. Intens Care Med. 2012;16;38(9):1523–31.
2. MacKinnon RJ, Slater D, Pukk-Härenstam K, von Thiele Schwarz U, Stenfors T. Adaptations to practice and resilience in a paediatric major trauma centre during a mass casualty incident. British journal of anaesthesia. 2022;1;128(2):e120-6.
3. Davis J, Marsh N. The myth of the universal child. Safeguarding Young People: risk, rights, resilience and relationships. Jessica Kingsley Publishers, London. 2022;21:111–28.

### P9. Extracorporeal cardiopulmonary resuscitation for out-of-hospital cardiac arrest: a retrospective observational study of pre-hospital patient eligibility in an urban area of Germany

#### Hanna Illian^1^, Clemens Grimm^1^, Nils Kunze-Szikszay^1^, Hanna Haus^1^, Claudius Balzer^1^, Markus Roessler^1^

##### ^1^Department of Anaesthesiology, University Medical Center Göttingen, Robert-Koch-Strasse 40, 37,075, Göttingen, Germany

**Correspondence:** Hanna Illian (hanna.illian@med.uni-goettingen.de)

*Scandinavian Journal of Trauma, Resuscitation and Emergency Medicine* 2024, **32(S1)**: P9

**Background** Out-of-hospital cardiac arrest (OHCA) is associated with a survival rate of less than 10% despite advances in treatment and technology [1]. Extracorporeal cardiopulmonary resuscitation (ECPR) can improve outcomes for select OHCA patients transported to a cardiac arrest center (CAC), providing extended circulatory support while reversible causes of arrest are treated [2, 3]. Our aim was to determine how many patients within the pre-hospital catchment area of our CAC, such as those with refractory ventricular fibrillation, could potentially benefit from ECPR.

**Materials and methods** This retrospective observational study utilised data from the German Resuscitation Registry, focusing on OHCA cases in Göttingen between 2009 and 2023. Patients (≥ 16 years) with non-traumatic cardiac arrest who received resuscitation efforts by pre-hospital emergency medicine were included. The criteria of the current operating procedure for ECPR at the University Medical Center Göttingen (UMG), including a no-flow time of ≤ 5 min, age < 75 years, and the absence of severe pre-existing conditions, were applied to assess the number and characteristics of potential ECPR candidates.

**Results** Of the 2083 OHCA cases 1,611 matched the general inclusion criteria. Following the application of the UMG criteria for ECPR eligibility, 221 patients were identified as potential candidates. Among the 221 patients analysed, the median age was 63 years, and 77.8% were male. Most arrests occurred at home (51.6%) or on the street (34.5%), with bystander-CPR in 61.5% (192) of the cases. Shockable rhythms were present in 47.5% of cases. 18.6% (41) patients were transported to the hospital under ongoing CPR. The mean transport time was 14.9 (interquartile range 8.7) minutes.

**Conclusion** There is a significant number of OHCA patients who meet our criteria for ECPR, resulting in approximately 15 eligible patients per year eligible for treatment in our CAC. The implementation of pre-hospital ECPR programs should be accompanied by well-designed clinical trials.

**References**

1. Yan S, Gan Y, Jiang N, Wang R, Chen Y, Luo Z et al. The global survival rate among adult out-of-hospital cardiac arrest patients who received cardiopulmonary resuscitation: a systematic review and meta-analysis. Crit Care. 2020;24(1):1–13. 10.1186/s13054-020-2773-2.
2. Ahn C, Kim W, Cho Y et al. Efficacy of extracorporeal cardiopulmonary resuscitation compared to conventional cardiopulmonary resuscitation for adult cardiac arrest patients: a systematic review and meta-analysis. Sci Rep. 2016;6(1):34,208. 10.1038/srep34208.
3. Twohig CJ, Singer B, Grier G, Davies G, Perkins ZB. A systematic literature review and meta-analysis of the effectiveness of extracorporeal-CPR versus conventional-CPR for adult patients in cardiac arrest. J Intensive Care Soc. 2019;20(4):347–357. 10.1177/1751143719832162.

### P10. Prehospital seizure management protocols need standardized guidelines. A descriptive study from Norway.

#### Ingrid Anette Hustad^1,2^, Morten Horn^1^. Marius Rehn^2,3,4^, Erik Taubøll^1,4^ and Maren Ranhoff Hov^1,2,5^

##### ^1^Dep. of Neurology, Oslo University Hospital; ^2^Department of Research and development, The Norwegian Air Ambulance Foundation. Oslo, Norway; ^3^Air Ambulance Department, Division of Prehospital Services, Oslo University Hospital, Oslo, Norway; ^4^ Institute of clinical medicine, University of Oslo, Postbox 1072, Blindern, Oslo, Norway; ^5^Faculty of Health Science, Oslo Metropolitan University

**Correspondence:** Ingrid Anette Hustad (ingrid.hustad@norskluftambulanse.no)

*Scandinavian Journal of Trauma, Resuscitation and Emergency Medicine* 2024, **32(S1)**: P10

**Background** Patients with convulsive seizures constitute a significant group in acute neurology. No common European clinical practice guidelines on prehospital seizure management exists, and today most patients are brought to hospital for seizure treatment, with great variation in which prehospital treatment is provided. Only 33% of status epilepticus patients receive a benzodiazepine as first anti-seizure medication (ASM). The aim of this study is to assess the prehospital seizure control protocols in the Emergency Medical Services (EMS) in Norway, and compare these with current evidence for acute management.

**Method** We performed a descriptive analysis of the 18 regional EMS protocols in Norway and compared the findings with recent evidence on prehospital treatment. We analysed recommended drug and dosage, route of medication administration, number of additional rescue doses permitted, requirements for registration of type of seizures and seizure duration.

**Results** The protocols vary in terms of preferred medication, administration method, dosage and recommendations regarding first- and second-line therapies. 33% of protocols explicitly define status epilepticus according to contemporary guidelines, and 16.7% have an operational definition of *when* to administer benzodiazepines. All protocols showed variations in dosing and administration instructions and only 28% had a clearly stated first line treatment.

**Conclusion** There are disparities in the prehospital seizure management protocols within the Norwegian healthcare system, a system comparable to other European countries. To improve seizure management there is a need for standardised guidelines for prehospital treatment.

Words: 235

### P11. Subclavian line insertion in a HEMS setting: a service evaluation

#### Sarah Morton^1^, James Mullet^1^, Karen Rhodes^1^ and Matt O’Meara^1^

##### ^1^Essex and Herts Air Ambulance Trust, Essex, UK

**Correspondence:** Sara Morton (sarah.morton@ehaat.org)

*Scandinavian Journal of Trauma, Resuscitation and Emergency Medicine* 2024, **32(S1)**: P11

**Background** Subclavian lines are useful in the acute setting for obtaining large bore vascular. Anecdotally clinicians experience difficulties, particularly in the prehospital Helicopter Emergency Medical Service (HEMS) setting. The aim of this service evaluation was to establish the incidence of subclavian line insertion in a busy mixed rural-urban UK HEMS service, the success rates and potential reasons for difficulties to improve future practice.

**Materials and Methods** A retrospective review of the anonymised computerised record system was performed between February 2016-April 2024 to identify patients who had a subclavian line insertion or attempt at insertion recorded in their notes (either documented as an intervention or described in a free text). Data was extracted relating to demographics, injury burden, success, number of attempts, complications and reasons for failure. Local research approval was gained.

**Results** 107 subclavian lines were attempted over an eight year period. Of those, 67 were successfully inserted (62.6%), with the majority of successes on the right side (n = 40, 60%). 57 of the successful insertions were inserted on the first attempt, with only one requiring three attempts. Success rates were higher for consultants (72%, 47 out of 65 attempted) than registrars (48%, 20 out of 42 attempted). Of the unsuccessful attempts (n = 40), the majority were abandoned after one failed attempt (n = 20). Reasons for difficulties noted included body habitus (n = 3), difficult landmarks due to injuries (n = 3), difficulty obtaining flashback (n = 7), an inability to advance the wire or cannula (n = 7) and poor positioning on a scoop (n = 1).

**Conclusion** Whilst a relatively rare intervention (approximately one per month), the success rate remains at around two thirds and therefore a good option if urgent vascular access is required. Injuries may limit the ability to identify landmark and positioning should be optimised to minimise difficulties.

### P12. Impact of the implementation of a networked-ROTEM system on code red trauma management at the Royal London Hospital

#### Suzanne Body^1^, Mattia Baldini^1^, Justin Maini^1^, Rebekah Holubinka^1^, Dan Nevin^1^

##### ^1^Department of Anaesthesia, Royal London Hospital, London, UK

**Correspondence:** Suzanne Body (S.body@nhs.net)

*Scandinavian Journal of Trauma, Resuscitation and Emergency Medicine* 2024, **32(S1)**: P12

**Background** For over a decade, Rotational Thromboelastometry (ROTEM) has been utilized at the Royal London Hospital (RLH) via a single analyser in the theatre recovery area. In 2023, 7 ROTEM machines were installed in critical areas with networked software (GEMweb) enabling real-time monitoring of a ROTEM test from any trust desktop, facilitating immediate ROTEM-guided resuscitation and correction of trauma coagulopathy. We audited the system after appropriate implementation time to identify frequency and utility of the system change.

**Method** We collected data on all Code Red patients, focussing on those requiring immediate resuscitation as well as those that died within the first 24 h. From patient records and GEMweb database we collected information about mechanism of injury, whether a ROTEM was performed and the time it took to perform the first ROTEM test (‘door to ROTEM time’).

**Results** Between September 1st, 2022, and May 3rd, 2024, there were a total of 211 Code Red incidents at the RLH, 50% of which required immediate resuscitation and 13 (12.3%) died within 24 h. With the introduction of GEMweb, the use of ROTEM increased in Code Red patients (from 43.1 to 77.8%), in particular in those needing immediate resuscitation (from 56 to 88.3%) and in those dying from traumatic haemorrhage (from 25 to 88.9%). After the introduction of GEMweb 69% of samples were processed in ED and, despite a wide range, the ‘door to ROTEM time’ decreased by 38 min.

**Conclusions** After the introduction of GEMweb, the use of ROTEM in Code Red patients has increased, especially for those requiring immediate resuscitation. The median time for achieving the test decreased when the networked system was fully operational. There is still wide variation in the time it takes for patients to receive their first ROTEM test, which may be due to different patient subgroups and variations in clinician practice.

**Reference**

1. Brill JB, Brenner M, Duchesne J, Roberts D, Ferrada P, Horer T, Kauvar D, Khan M, Kirkpatrick A, Ordonez C, Perreira B, Priouzram A, Cotton BA. The Role of TEG and ROTEM inlShock. 2021 Dec 1;56(1S):52-61. 10.1097/SHK.0000000000001686. PMID: 33769424; PMCID: PMC8601668.

### P13

#### Robert S. Green^1^, Hillary Ferguson^1^, Izabella Opra^2^, Dan Cashen^2^, Adam Harris^3^, Mete Erdogan^2^

##### ^1^Department of Critical Care, Dalhousie University, Halifax, Nova Scotia, Canada, B3H 3A7, ^2^Trauma Nova Scotia, Nova Scotia Health, Halifax, Nova Scotia, Canada, B3H 2Y9, ^3^Department of Emergency Medicine, Dalhousie University, Halifax, Nova Scotia, Canada, B3H 3A7

Impact of a dedicated trauma consult service on burnout levels among physicians and nurses; a longitudinal survey

**Correspondence:** Robert S. Green (Robert.Green@nshealth.ca)

*Scandinavian Journal of Trauma, Resuscitation and Emergency Medicine* 2024, **32(S1)**: P13

**Background** Trauma providers have previously reported high levels of burnout attributed to various factors [1], which were exacerbated by the COVID-19 pandemic. As part of a novel redesign of the care delivery model of major trauma patients in the province of Nova Scotia, we sought to assess the impact of introducing a dedicated team approach to inpatient care (i.e., the Trauma Consult Service) on provider burnout levels at 6 and 12 months.

**Methods** A longitudinal electronic survey was developed using SelectSurvey and piloted by members of the research team. A baseline survey was administered in November 2022 to all TC Physicians, TC Nurses, and Trauma Team Leaders (TTLs). The survey collected demographic data and evaluated burnout symptoms using the Copenhagen Burnout Inventory (CBI), the Maslach Burnout Inventory (MBI) single-item Emotional Exhaustion (EE) and Depersonalization (DP) scales, and the Utrecht Work Engagement Scale (UWES). Follow-up surveys were administered at 6-months and 12-months. Generalized estimating equations (GEE) models were fitted to determine the effect of the TC Service on burnout symptoms during the inaugural year of service.

**Results** A total of 29 survey responses were received from TTL/TC Physicians (n = 13), TTLs (n = 9) and TC Nurses (n = 7). Nearly all were full-time employees (96.6%) and most had 6-10 years experience providing trauma care (58.6%). Few participants reported using mental health resources to improve their wellbeing (3.4%). At 1-year follow-up, there were reductions in CBI scales measuring personal burnout (OR 0.93, 95% CI 0.89-0.97), work-related burnout (OR 0.93, 95% CI 0.87-0.99), and in the MBI single-item measures for EE (OR 0.88, 95% CI 0.79-0.98) and DP (OR 0.86, 95% CI 0.76-0.98). GEE models revealed increased staff engagement across all 3 UWES scales.

**Conclusion** Burnout symptoms among Physicians and Nurses were significantly reduced at 1-year post-implementation of a dedicated inpatient TC Service.

**Reference**

1. de Wit K, Tran A, Clayton N, Seeburruth D, Lim RK, Archambault PM, Chan TM, Rang LCF, Gray S, Ritchie K, Gérin-Lajoie C, Mercuri M; Network of Canadian emergency researchers. A longitudinal survey on Canadian emergency physician burnout. Ann Emerg Med. 2024; 83:576–584.

### P14. Key performance indicators in major trauma haemorrhage: developing best practice for ‘code red’ trauma

#### Manik Chana^1,2^, Hayley Evans^3^, Akshay Shah^4^, Dan Frith^1^, Robbie Foy^5^, Simon Stanworth^3^

##### ^1^Traum@IC Research Group, St. Mary’s Major Trauma Centre, Imperial College London; ^2^The Nuffield Division of Clinical Laboratory Sciences, Division of Medical Sciences, University of Oxford; ^3^NIHR Blood and Transplant Research Unit in Data Driven Transfusion Practice, Radcliffe Department of Medicine, University of Oxford, Oxford, UK; ^4^Nuffield Department of Clinical Neurosciences, University of Oxford, Oxford, UK; ^5^Leeds Institute of Health Sciences, University of Leeds, Leeds, UK

**Correspondence:** Manik Chana (manik.chana@ndcls.ox.ac.uk)

*Scandinavian Journal of Trauma, Resuscitation and Emergency Medicine* 2024, **32(S1)**: P14

**Background:** Major haemorrhage following trauma accounts for approximately 50% of the 4.6 million annual injury related deaths worldwide. In developed trauma systems, the initial management of these patients is based on balanced transfusions of blood and blood products with tranexamic acid. Major Haemorrhage Protocols (MHPs) formalise the process, personnel and products to be delivered in an efficient manner. They are resource intensive and place demand on healthcare institutions. Despite international guidelines, there is currently no agreed criteria for activation of a MHP, and no agreed measure of the appropriateness or success of their use. Key Performance Indicators (KPIs) can be broadly defined as validated measures of care. We aimed to develop a set of evidence based KPIs for major trauma haemorrhage utilising routinely collected electronic health data.

**Methods:** A scoping review of literature was performed for transfusion in major trauma from 2000 to 2023. Additionally, a number of Major Trauma Centres in England were approached for their adult MHP used in trauma. A list of KPIs was generated from these studies, then refined to focus on parameters which could be collected from routinely collected electronic data.

**Results: **After screening, 35 studies were included from the literature along with 8 institutional MHPs. This yielded 15 potential KPIs. These pertain to activation of MHP, targets for transfusion, product ratios and wastage.

**Discussion: **Currently there is no agreed measure of the appropriateness or success of transfusion in major trauma haemorrhage. Whilst KPIs cannot form guidelines, they provide a metric on which to measure adherence to best practice and reduce unwanted variance. This process will undergo further validation of the feasibility and clinical importance of identified KPIs, as well as field testing. Establishing validated KPIs will help to develop the gold standard in care of the ‘ Code Red’ patient.

### P15. Paediatric trauma resuscitation course–a novel approach to multidisciplinary training improving confidence in treatment of paediatric trauma cases

#### Julia Dumesh^1^, Fiona Lyle^2^, Rob Hirst^2^, Chris Hook^1^, Gavin Wooldridge^1^, Paul Reavley^1^

##### ^1^Department of Emergency Medicine, Bristol Royal Infirmary, University Hospitals Bristol & Weston, Bristol, UK, ^2^Department of Emergency Medicine, Bristol Royal Hospital for Children, University Hospitals Bristol & Weston, Bristol, UK

**Correspondence:** Julia Dumesh (paedtraumaresuscourse@gmail.com)

*Scandinavian Journal of Trauma, Resuscitation and Emergency Medicine* 2024, **32(S1)**: P15

**Background** This novel course inspired by the work of the Paediatric Blast Injuries Partnership (PBIP) between Imperial College London & Save the Children UK, focuses on a unique simulation-based approach to paediatric trauma training.

**Method** Through the use of interactive simulation scenarios adapted to fit a variety of contexts and resources, this course builds the confidence of healthcare professionals to in-hospital treatment of the paediatric trauma patient. Multiple scenarios of potential paediatric trauma injuries are presented for multidisciplinary team management, and feedback collected from delegates about their confidence levels in treating these injuries before & after completion of the course.

**Results **39 questionnaires filled out by delegates on the course have demonstrated improved confidence levels in their approach to paediatric trauma cases after this course. Delegates reported feeling ‘not at all confident’ or only ‘slightly confident’ when presented with twenty potential trauma cases prior to the course. This significantly improved to feeling fairly or completely confident by the end of the course, in some scenarios with zero delegates reporting ongoing lack of confidence.

**Conclusion **More children live in conflict zones at this time than any other in the current millennium and in many areas worldwide, trauma is the leading cause of child death. Everyday more clinicians around the world are faced with the challenge of how to treat severely injured children. [1] There is a current lack of global training resources for healthcare professionals to treat paediatric traumatic injuries when compared with the many resources for paediatric medicine and for adult trauma training. [2] This course provides a supportive multidisciplinary learning environment to target this gap in existing training resources. The authors aim to continue this work to provide the course free of charge to all, in as many trauma networks as possible.

**References**

1. Wild H, Reavley P, Mayhew E, Ameh EA, Celikkaya ME, Stewart B. Strengthening the emergency health response to children wounded by explosive weapons in conflict. World J Pediatr Surg. 2022 Aug;5(4):e000443.
2. Hamill J, Beasley SW. Training in paediatric trauma: the problem of safer societies. ANZ J Surg. 2006 Jul;76(7):596–9.

### P16. Major trauma patients and services in the East of England Trauma Network, a decade after its establishment

#### Lauren Rixson^1^, Zidong Liu^2^, Houyuan Jiang^2^, Feryal Erhun^2^, Esther Kwong^1^

##### ^1^Public Health Directorate, NHS England – East of England, Cambridge CB21 5XB, UK; ^2^Judge Business School, University of Cambridge, Cambridge CB2 1AG, UK

**Correspondence:** Zidong Liu (zl424@jbs.cam.ac.uk); Lauren Rixson (lauren.rixson@nhs.net), Houyuan Jiang(h.jiang@jbs.cam.ac.uk), Feryal Erhun(f.erhun@jbs.cam.ac.uk), Esther Kwong (esther.kwong1@nhs.net)

*Scandinavian Journal of Trauma, Resuscitation and Emergency Medicine* 2024, **32(S1)**: P16

**Background** Since 2013, the East of England Trauma Network (EoE TN) has seen a steady rise in trauma patients, yet its service capacity, particularly trauma bed availability, has not been reassessed in over a decade. With one Major Trauma Centre (MTC) and multiple Trauma Units (TUs), this study evaluated if the existing infrastructure can meet reported changing demographics and rising trauma admissions.

**Methods** We analysed nine years (2013-2021) of Trauma Audit and Research Network (TARN) data, evaluating bed occupancy, service outcomes (length of stay, survival rates, Glasgow Outcome Score (GOS)), and patient pathways (Direct-to-MTC, Transfer-to-MTC, Direct-to-TU). Population trends were analysed using joinpoint regression by age, sex, injury mechanism, and Injury Severity Score (ISS), with future demand projected using an ARIMA model.

**Results** MTC bed occupancy has steadily exceeded capacity since 2018, coinciding with the rise in trauma cases. The age-adjusted rate of major trauma increased by 4.7% annually, with the highest growth (7.7% per year) in patients over 75 years. By 2026, trauma admissions are projected to rise by one-third. Falls less than two metres had the highest rise (8.8% per year), contributing to the increased burden. Despite the growth in major trauma, 62.5% of major trauma cases were managed in TUs, with lower MTC access for women, elderly patients, and those from deprived areas. While Direct-to-TU patients had lower ISS, their GOS outcomes were comparable to Direct-to-MTC patients.

**Conclusion** Increasing trauma demand, particularly among older patients and those with falls, combined with MTC capacity limits, necessitates changes in the EoE TN. Equitable access and appropriate management across TUs and the MTC are critical as the network adapts to growing needs. Further research on care distribution and access disparities is essential.

**Acknowledgements** We thank Mr Matt Targett and Dr Corinna Pascuzzi from EoE Trauma Network.

### P17. Proof of concept for a novel non-compressible haemorrhage control device

#### Nicky Johnson^1^, Joseph Bentley^1^, Emma Priestley^1^, Nathan Wenban^1^

##### ^1^ACT Medical Ltd, Cambridge, UK

**Correspondence:** Nicky Johnson (hello@actmedical.co.uk)

*Scandinavian Journal of Trauma, Resuscitation and Emergency Medicine* 2024, **32(S1)**: P17

**Background **The management of extremity wounds with arterial bleeding has been revolutionised by the tourniquet. Intense efforts have been made worldwide to develop effective treatment for junctional haemorrhagic wounds that cannot be addressed with tourniquets or are non-compressible. However, existing market solutions still rely on manual compression and are challenging to apply to profusely bleeding intracavity wounds. Haemostatic agents embedded into gauze carry risks such as embolic phenomena, difficult deployment, and incomplete agent retrieval [1–3].

**Method **This study utilised three live adult porcine (1 female and 2 castrated males) to demonstrate the safety profile of a tamponade-based device to the market standard treatment, ensuring that ACT Medical’s device did not pose a higher risk of adverse events.

**Results **Catastrophic haemorrhage control was achieved at three minutes for all six injuries treated with ACT Medical’s device. The safety profile was confirmed, with no adverse events reported. Post treatment visual blood loss was significantly less compared with the market comparator, and total application time for treatment was also significantly less in ACT Medical’s device.

**Conclusion** Application of direct internal pressure is an effective means of haemorrhage control which could reduce post treatment blood loss and application time. Once undergoing damage control surgery, the device can be removed in one piece, with minimal damage to the wound bed. Further research is needed to verify the safety and efficacy of ACT Medical’s device.

**References**

1. Donley ER, Munakomi S, Loyd JW. Hemorrhage control. Available from: https://www.ncbi.nlm.nih.gov/books/NBK535393/ (Accessed 2024 Jun 04).
2. Khoshmohabat H, Paydar S, Mohammad Kazemi H, Dalfardi B. Overview of agents used for emergency hemostasis. Trauma Monthly. 2016;21(1):e26023. Available from: https://www.ncbi.nlm.nih.gov/pmc/articles/PMC4869418/ (Accessed 2024 Aug 11).
3. Bennett BL. Bleeding control using hemostatic dressings: lessons learned. Wilderness Environ Med. 2017;28(2):S39-S49.

### P18. Establishing the safety of emergency department delivered regional anaesthesia for rib fractures

#### Kuroush Ardeshirian^1^, Gokul Sagar^1^, Gabriel Jones^1^, Harriet Tucker^1^, Anthony Hudson^1^

##### ^1^St George’s Hospital Emergency Department, London, UK

**Correspondence:** Kuroush Ardeshirian (k.ardeshirian@gmail.com)

*Scandinavian Journal of Trauma, Resuscitation and Emergency Medicine* 2024, **32(S1)**: P18

**Background** Blunt chest trauma accounts for up to 15% of all trauma admissions to emergency departments (EDs) worldwide, with mortality of 4–60% from complications including pneumonia and respiratory failure [1]. Adequate early analgesia has a key role in complication prevention, however opioids can cause unfavourable side effects [2]. Fascial plane regional anaesthesia, such as serratus anterior (SAPB) and erector spinae plane blocks (ESPB), provide effective and safe analgesia for patients with high-risk rib fractures [2]. In the United Kingdom, these are typically inserted by anaesthetic teams on emergency theatre lists; however local audits found long delays to insertion (3–5 days). Therefore we aimed to establish a safe ED-delivered regional anaesthesia programme for patients with high-risk rib fractures.

**Method **At this London Major Trauma Centre a thoracic wall injury standard operating procedure was created. Patients with rib fractures were stratified into low or high risk of complications by Battle’s score. High risk patients subsequently received ED-delivered regional anaesthesia (SABP or ESBP), with or without a catheter.

**Results **220 regional blocks were performed over 30 months (151 ‘single-shot’ blocks and 69 catheters). There were 23 complications (10%). 18 (8%) of these were catheter-related (dislodgement, disconnection or occlusion). 2 (0.9%) of the catheters displayed localised signs of infection. These were removed but required no further intervention. Importantly, there was one case of a wrong-sided block following a typographical error in a provisional radiology report.

**Discussion **The majority of complications involved equipment (catheter-related) malfunction, and most required reinsertion by anaesthetic teams. However, this ED-placed catheter secondary failure rate of 26 per cent is lower than the 41 per cent found in a concurrent audit of all regional catheters at the same institute. There were minimal infection, and no bleeding-related complications.

This project demonstrates the safety of ED-delivered regional anaesthesia for patients with high-risk rib fractures.

**References**

1. Battle C, Hutchings HA, Driscoll T, O’Neill C, Groves S, Watkins A, Lecky FE, Jones S, Gagg J, Body R, Abbott Z. A multicentre randomised feasibility study evaluating the impact of a prognostic model for management of blunt chest wall trauma patients: STUMBL trial. BMJ Open. 2019 Jul 1;9(7):e029187.
2. Adhikary SD, Liu WM, Fuller E, Cruz‐Eng H, Chin KJ. The effect of erector spinae plane block on respiratory and analgesic outcomes in multiple rib fractures: a retrospective cohort study. Anaesthesia. 2019 May;74(5):585–93.

### P19. Impact of response times on outcomes in UMMC pre-hospital care for road traffic accidents: a six-month analysis within Klang Valley

#### Afiq Mohd Nor^1^, Salleh Yahya^1^

##### ^1^Emergency Medicine Department, University Malaya Medical Centre, Kuala Lumpur, Malaysia; ^2^Emergency Medicine Department, Faculty of Medicine, University of Malaya, Kuala Lumpur, Malaysia

**Correspondence:** Afiq Mohd Nor (afiq@ummc.edu.my)

*Scandinavian Journal of Trauma, Resuscitation and Emergency Medicine* 2024, **32(S1)**: P19

**Background** Response times in pre-hospital care are critical for determining outcomes in road traffic accidents (RTAs), especially for high-priority cases [1]. This study analyses the relationship between priority level calls, response times, dispatch times, and patient outcomes in motor vehicle accidents (MVAs) within the Klang Valley region, focusing on cases transported to a tertiary teaching hospital, Universiti Malaya Medical Centre (UMMC) during the first half of 2024.

**Method **A retrospective analysis was conducted on 220 MVA cases managed by UMMC pre-hospital emergency services from January to June 2024. Response times, dispatch times, and patient outcomes were extracted from ambulance call logs. Outcomes were categorised by triage levels (T1, T2, T3) and analysed for correlations with response times. Statistical analysis explored the significance of response times to patient outcomes, using logistic regression to model the impact on mortality. Compliance issues in dataset documentation were also evaluated.

**Results **UMMC received the majority of T1 cases (n = 33). The average response time across all cases was 7.93 min, with T1 patients averaging 12.55 min. In Priority 1 cases, patients who died had a response time of 20.83 min compared to 13.10 min for other outcomes. A t-test indicated a statistically significant difference (*p* = 0.0136), indicating that delayed response times correlate significantly with increased mortality. Logistic regression showed that each minute of delayed response time increased the odds of mortality (coefficient = 0.0711, *p* = 0.019). Documentation revealed forty-nine missing hospital destination entries, twelve missing AT-SITE TIME logs, and three cases with negative time intervals, indicating recording data compliance issues.

**Conclusion **Delays in response times are significantly associated with increased mortality in high-priority MVA cases [2]. Logistic regression further highlights the critical role of rapid response. Addressing response time inefficiencies and improving data documentation is crucial to enhancing patient care and the reliability of future analysis.

**References**

1. Ito, S., Asai, H., Kawai, Y. et al. Factors associated with EMS on-scene time and its regional difference in road traffic injuries: a population-based observational study. BMC Emerg Med 22, 160 (2022). 10.1186/s12873-022-00718-1.
2. Waalwijk JF, van der Sluijs R, Lokerman RD, Fiddelers AAA, Hietbrink F, Leenen LPH, Poeze M, van Heijl M; Pre-hospital Trauma Triage Research Collaborative (PTTRC). The impact of pre-hospital time intervals on mortality in moderately and severely injured patients. J Trauma Acute Care Surg. 2022 Mar 1;92(3):520–527. 10.1097/TA.0000000000003380. PMID: 34,407,005.

### P20. Development of the post-ATLS perioperative polytrauma protocol at the Royal Sussex County Hospital—the ‘4P’ protocol

#### Mark Parson^1^

##### ^1^The Royal Sussex County Hospital, Easton Road, Brighton

**Correspondence:** Mark Parson (mark.parson2@nhs.net)

*Scandinavian Journal of Trauma, Resuscitation and Emergency Medicine* 2024, **32(S1)**: P20

**Background** At the Royal Sussex County Hospital (RSCH), delays in admitting patients from A&E to the wards, particularly those who have undergone initial ATLS-based resuscitation for trauma, have been identified as a contributor to suboptimal care prior to surgery. Local Morbidity and Mortality meetings revealed that this patient cohort often faces environmental and organizational challenges, as well as conflicting resuscitation paradigms. In response, the Major Trauma Centre team recognized the need for an evidence-based, unified protocol to streamline post-ATLS perioperative care for polytrauma patients, particularly those with femoral and/or pelvic fractures. This led to the development of the '4P Protocol' to guide Multi-Disciplinary Team (MDT) management.

**Methods** A comprehensive literature search was conducted by Sussex Health Knowledge and Libraries, focusing on guidelines for the management of polytrauma patients following initial resuscitation. The evidence was compiled into a table, and an MDT of clinicians reviewed the findings.

**Results** The overarching goals of the protocol are to optimize patients for surgery by [1]:

- Preventing coagulopathy,
- Warming the patient,
- Continuing volume resuscitation,
- Preventing electrolyte imbalances.

The protocol recommends definitive fixation within 36 h for resuscitated patients meeting at least one of the following criteria [2]:

- Venous lactate < 4.0 mmol/L,
- Base excess (BE) ≥  − 5.5 mmol/L,
- pH ≥ 7.25.

To achieve these resuscitation targets, the protocol includes the following guidelines:

- Catheterization with urine output > 0.5 mL/kg/hr,
- Thresholds for packed red cells, fresh frozen plasma, platelet, and cryoprecipitate transfusions,
- Viscoelastic-based goal-directed resuscitation for massive transfusion patients,
- Minimization of crystalloid fluid administration.

These clinical considerations are consolidated into a flow diagram to ensure streamlined and consistent patient management.

**Conclusion** An agreed-upon protocol has been developed with input from key stakeholders at RSCH. We invite feedback from clinicians at the London Trauma Conference to refine the protocol further before implementation.

References

1. Keane M. Triad of death: the importance of temperature monitoring in trauma patients. Emergency Nurse. 2016 Sep 12;24(5).
2. Vallier HA, Moore TA, Como JJ, et al. Complications are reduced with a protocol to standardize timing of fixation based on response to resuscitation. J Orthop Surg Res. 2015;10:155. Published 2015 Oct 1. 10.1186/s13018-015-0298-1.

### P21. The bleep test: a table-top simulation to develop clinical reasoning and resource management for anaesthetic trainees

#### Mark Parson^1^

##### ^1^St. George’s University of London, London, UK

**Correspondence:** Mark Parson (mark.parson2@nhs.net)

*Scandinavian Journal of Trauma, Resuscitation and Emergency Medicine* 2024, **32(S1)**: P21

**Background** Tabletop Simulation (TTX) is an rapidly developing field in medical education that prioritizes Crisis Resource Management (CRM) skills over technological or environmental fidelity [1]. To date, the majority of TTX has been implemented in disaster healthcare [2], however this is changing [3]. In response to a deficit of CRM training for overnight on-call staff at The Royal Sussex County Hospital in Brighton, an anaesthetic TTX called "The Bleep Test" was developed. "The Bleep Test" places players in a fictional hospital setting with limited deployable resources. Each turn presents resource demands in the form of tasks, requiring players to prioritize based on situational constraints and clinical needs. The game’s goal is not to win but to enhance crisis resource management skills through structured facilitation.

**Methods **Two educational sessions were conducted, each lasting one hour, with three games running concurrently. Each game was facilitated by a senior anaesthetic clinician. After completing the game, anaesthetic trainees were surveyed on their experience.

**Results **11.8% (n = 2) trainees had had training for on-call crisis resource management. 100% (n = 17) of trainees gave the game ≥ 4 out of 5 for both 1) How useful, and 2) how enjoyable the learning exercise was. 100% (n = 17) would play it again. Freetext comments included “Incredibly fun game and great way to tease out problem solving and non-clinical skills required on a typical “busy” night shift!” and “good springboard for discussions about the management of anaesthetic emergencies”. Most commonly cited free text improvement was improving the rule book clarity (23.5%; n = 4). All games were started and completed within the one-hour time frame.

**Conclusion **"The Bleep Test" successfully addresses a training gap in on-call CRM. While further evaluation is needed to assess long-term impacts on confidence and skill development, the game has proven to be an engaging and effective short-term educational tool.

**References**

1. Frégeau A, Cournoyer A, Maheu-Cadotte MA, et al. Use of tabletop exercises for healthcare education: a scoping review protocol. BMJ Open. 2020;10(1):e032662. Published 2020 Jan 7. 10.1136/bmjopen-2019-032662.
2. Evans CA, Schwartz R. Using tabletop exercises as an innovative and practical teaching strategy in response to external disaster scenarios. Nurs Educ Perspect. 2019;40(1):62–64.
3. Sanko J. How to use table-top simulation games as part of simulation-based education [Internet]. Healthysimulation.com. 2023 [cited 2024 Jul 27]. Available from: https://www.healthysimulation.com/52915/table-top-healthcare-simulation/.

### P22. Severe multiple trauma in patients from suicide attempts: a treatment analysis in single center of South Korea

#### Yejune Pae^1^, Han-Young Lee^1^, Jae-myeong Lee^1^

##### ^1^Division of Acute Care Surgery, Department of Surgery, Korea University Anam Hospital, Korea University Medical Center, Seoul, Republic of Korea

**Correspondence:** Jae-myeong Lee (ljm3225@hanmail.net)

*Scandinavian Journal of Trauma, Resuscitation and Emergency Medicine* 2024, **32(S1)**: P22

**Background** The high suicide rate remains a pressing issue in Korea. While numerous studies have explored factors related to suicide attempts, there is a lack of research on the recovery process following severe trauma after suicidal attempts. This study aims to analyze severe trauma from suicide attempts, such as suicidal methods, mortality rates, degree of injury, and recovery outcomes. We hypothesize that falls will lead to greater physical impacts, resulting in higher fatality rates and treatment requirements, particularly for fractures in critical areas such as the spine or pelvis.

**Methods** This study examined patients admitted to the Severe Trauma Final Treatment Center at Korea University Anam Hospital in Seoul, Korea, over a two-year period from January 2021 to December 2022. Only cases determined to be suicide attempts were included. We compared the characteristics of deceased patients and documented their psychiatric histories based on treatments received in the emergency room or during hospitalization.

**Results** The mortality rate was significantly higher in patients without a prior psychiatric diagnosis compared to those with a diagnosed mental disorder (33.3% vs. 5.8%, *p* = 0.016). Women experienced more falls (68.2% vs. 41.3%, *p* = 0.010), while men were more likely to use stabbing methods (26.1% vs. 9.1%, *p* = 0.035). The distribution of stab wound locations for men was: abdomen (64%), neck (29.4%), chest (5.9%), and wrist (0%); for women, it was: abdomen (40%), wrist (30%), and neck (30%). The highest mortality rates were associated with fractures in the following sites: upper limb (60%), skull (45.5%), pelvic bone (34.8%), rib (34.4%), facial bone (30.0%), lower limb (29.6%), and spine (18.8%). Increased fall height correlated with higher mortality (*p* = 0.048), although survivors were noted from falls over 30 m (about 13 stories) when landing on softer surfaces such as wires (N = 1), trees (N = 1), and the Han River (N = 1).

**Conclusion** Mortality rates were significantly higher for suicide attempts associated with acute stress or untreated psychiatric disorders. Women displayed a higher incidence of wrist cuts, typically with shallow penetration. Interestingly, mortality was lowest in cases involving spine fractures. Additionally, higher falls led to increased mortality, although a cushioning effect from certain surfaces appeared to lower mortality rates.

### P23. Pre-hospital hyperglycaemia in traumatic brain injury is associated with mortality and poorer functional outcomes at six months: a single centre retrospective observational study

#### Joseph D Wilson^1^, Jack Barrett^2^, Jo Griggs^2^, Malcolm Tunnicliff^1,2^

##### ^1^King’s College Hospital, London; ^2^Air Ambulance Charity Kent Surrey Sussex

**Correspondence:** Joseph D Wilson (joseph.wilson10@nhs.net)

*Scandinavian Journal of Trauma, Resuscitation and Emergency Medicine* 2024, **32(S1)**: P23

**Background** Traumatic brain injury (TBI) is a major contributor to mortality constituting 40% of all injury-related deaths worldwide [1]. Following primary brain injury, there is rapid onset of a secondary brain injury underpinned by vascular and metabolic cascades [2]. Hyperglycaemia, within this secondary injury, drives poor functional and mortality outcomes; consequently tight glycaemic control is a cornerstone of neurocritical care in TBI patients [3]. There are no guidelines for the pre-hospital management of hyperglycaemia in patients with suspected TBI. We hypothesise that pre-hospital hyperglycaemia in TBI patients is associated with poorer outcomes.

**Methods** 311 patients with clinical suspicion for TBI were transferred by Kent Surrey Sussex Air Ambulance to King’s College Hospital between January 2021 and August 2023. Inclusion criteria were: radiological evidence of TBI; a recorded pre-hospital glucose concentration; neurosurgical follow up at 6 months. Patients were excluded if < 18 years old. Functional deficits according to the Glasgow Outcome Score (GOS) were inferred from neurosurgical clinic letters.

**Results** 54 patients were included. Hyperglycaemia was associated with poorer functional outcomes at 6 months, yet did not reach statistical significance (*p* = 0.097). The fitted regression model was: GOS = 3.54 − ([glucose] × 0.11); there was substantial variation within the model (R^2^ = 0.05). Patients who did not survive to 6 months (n = 24) had a higher glucose concentration (8.50 mmol/L vs 8.00 mmol/L), yet did not reach statistical significance (*p* = 0.161). There was no difference in glucose concentration between severe (n = 40), moderate (n = 9), and mild (n = 5) TBI when stratified by initial GCS.

**Discussion** We demonstrate a trend between pre-hospital hyperglycaemia and increased mortality and morbidity in TBI patients. The lack of difference between glucose concentration within TBI groups stratified by severity implies that pre-hospital glucose concentration is not a surrogate marker for primary brain injury. These relationships require further discussion and interrogation given its importance in neurocritical care.

**References**

1. Maas AI, Menon DK, Adelson PD, Andelic N, Bell MJ, Belli A, et al. Traumatic brain injury: Integrated approaches to improve prevention, clinical care, and research. Lancet Neurology. 2017 Dec;16(12):987–1048. 10.1016/s1474-4422(17)30371-x.
2. Mckee AC, Daneshvar DH. The neuropathology of traumatic brain injury. Handb Clin Neurol. 2015;127:45–66. 10.1016/B978-0-444-52892-6.00004-0.
3. Yuan T, He H, Liu Y, Wang J, Kang X, Fu G, Xie F, Li A, Chen J, Wang W. Association between blood glucose levels and Glasgow Outcome Score in patients with traumatic brain injury: secondary analysis of a randomized trial. Trials. 2022 Jan 15;23(1):38. 10.1186/s13063-022-06005-5.

### P24. A service evaluation of the interventions performed in paediatric patients attended by an enhanced pre-hospital care team

#### Kalsoom Adil^1^

##### ^1^School of Medical Sciences, University of Manchester, Manchester, United Kingdom

*Scandinavian Journal of Trauma, Resuscitation and Emergency Medicine* 2024, **32(S1)**: P24

**Background** Trauma is the leading of cause of death in paediatric patients globally [1]. Interventions such as intubation in paediatric patients have been well documented however there is little current evidence regarding the prehospital critical care provided to paediatric patients by an Enhanced Pre-Hospital Care (EPHC) team. The aim of this service evaluation was to systematically analyse the interventions performed in paediatric patients attended to by the North West Air Ambulance (NWAA) EPHC team.

**Method** In this retrospective study, the electronic database (HEMSbase) was searched in a 5-year timeframe to analyse the critical care interventions performed in paediatric patients aged 0-16 years.

**Results** Overall, the case notes of 417 paediatric patients were analysed. The most common clinical presentation in these patients was road traffic collisions (35.7%) followed by accidental injury (24%). 18.9% of patients received endotracheal intubation. Blood transfusions were given to 5.8% of patients. Of these, 75% of patients were intubated and 4.2% had continuous temperature monitoring using an oesophageal temperature probe. 5.5% of patients underwent an open thoracostomy and 1% of patients underwent a resuscitative thoracotomy.

**Conclusion** The results of this study suggest critical care interventions such as blood transfusions, open thoracostomy and resuscitative thoracotomy are performed infrequently in paediatric patients attended to by the NWAA EPHC team. This reinforces the need for regular educational training for the EPHC team to maintain their skills and prevent clinician anxiety in performing these critical care interventions. In addition, further exploration of active warming measures such as warmed blood and fluid transfusions is required to combat the risk of hypothermia in paediatric patients. Further research into these critical care interventions is necessary, particularly in the paediatric subset.

**Reference**

1. Nesje E, Valøy NN, Krüger AJ, Uleberg O. Epidemiology of paediatric trauma in Norway: a single-trauma centre observational study. Int J Emerg Med. 2019; Jul 31;12(1).

### P25. The pre-hospital management of acute behavioural disturbance: a systematic literature review

#### Fredrick J.E Smith^1^, Jennifer Todd^2^, Pascale Avery^3^, Sarah Morton^4^

##### ^1^Epsom and St Helier University Hospital NHS Trust, London, UK; ^2^Guy’s and St Thomas’ NHS Foundation Trust, London, UK; ^3^North Bristol NHS Trust, Bristol, UK; ^4^Imperial College Healthcare NHS Trust, London, UK

**Correspondence:** Fredrick J.E Smith (f.smith26@nhs.net)

*Scandinavian Journal of Trauma, Resuscitation and Emergency Medicine* 2024, **32(S1)**: P25

**Background and objectives** Acute behavioural disturbance (ABD) is an under recognised condition that exists at the severe end of the spectrum of agitation. The condition is dangerous for both patients and providers and has several different causes including substance intoxication, mental health and occasionally medical conditions. Trauma can also cause or co-exist with ABD. Our objective was to systematically review the current evidence for the management of ABD in the pre-hospital setting.

**Methods** A systematic literature search (PROSPERO CRD42023447238) of PubMed, Cochrane trials, Cochrane reviews, Embase, Web of knowledge, Google Scholar and MEDLINE was performed from inception until September 2023. Any study that examined the pre-hospital management of ABD was included. Randomised controlled trials (RCTs), observational cohort studies and case series written in English were included. Methodological quality of included studies was interpreted using the Cochrane risk of bias tool, and rated using the Grading of Recommendations Assessment, Development, and Evaluation (GRADE) approach.

**Results** From 5385, 42 studies were included; one of moderate quality (n = 1), the rest were of low (n = 15) or very low quality (n = 26). Ketamine demonstrated the most effective sedation with 79-98% of patients included achieving adequate sedation, although doses and methods of administered varied significantly. Midazolam generally showed a higher number of side effects than other drugs studied. Droperidol was not shown to have any higher mortality, and no effect was seen on the QT interval in studies where it was examined.

**Conclusion** Ketamine is the most examined drug for treatment of ABD in the pre-hospital setting and is likely the most effective method of sedation. Midazolam appears to have a higher risk of side effects that are largely respiratory, and airway related. Additional research is required into the most effective mode of administration and dose for this patient population. Finally, droperidol is underutilised likely due to unfounded concerns surrounding torsade’s de points.

### P26. Improving follow-up care for patients with isolated rib fracture: a pilot study in a level 1 trauma centre

#### Talia Sener^1^, Guy Hans^2^, Suresh Krishan Yogeswaran^3^, Brigitte Claes^4^, Phara Elsen^4^, Sabine Lemoyne^5^, Philip Verdonck^5^

##### ^1^Faculty of Medicine and Health sciences, University of Antwerp, Antwerp, Belgium; ^2^Multidisciplinary pain centre, Antwerp University Hospital, Edegem, Belgium; ^3^Thoracovascular surgery department, Antwerp University Hospital, Edegem, Belgium; ^4^Digital outpatient follow-up service, Antwerp University Hospital, Edegem, Belgium; ^5^Emergency department, Antwerp University Hospital, Edegem, Belgium

**Correspondence:** Talia Sener (taliasener@icloud.com)

*Scandinavian Journal of Trauma, Resuscitation and Emergency Medicine* 2024, **32(S1)**: P26

**Background** Isolated rib fractures are common in emergency departments (ED) and require analgesia with outpatient follow-up. Poor pain management can cause complications. Early identification of inadequate analgesia, impaired breathing and signs of pneumonia are crucial. We explored the feasibility of a digital outpatient care pathway for patients with isolated rib fractures to enhance follow-up compliance.

**Methods** This monocentric study included patients (> 18 years) with isolated rib fractures, who could use a smartphone. Patients were excluded if admission was needed for associated injuries or analgesia. Eligible patients downloaded the hospital app and connected a pulse oximeter via Bluetooth. The digital pathway involved standardized daily surveys on pain (Visual Analogue Scale (VAS) score), analgesia adherence, breathing (with an incentive spirometry) and oxygen saturation, completed every other day during week 1, and weekly for 6 weeks. Two video consultations with pain specialists were scheduled at week 4 and 6, followed by a final consultation with a chest X-ray by a thoracic trauma surgeon at week 8.

**Results** From July to September 2024, 12 patients were included (mean age 59 years, range 39–84, 66% female). Four dropped out: 1 due to a suicide attempt, 1 due to smartphone issues, 1 due to technology failure and 1 refused follow-up. Of the remaining 8 patients, 5 completed all surveys, while 3 completed 6 of 10 total questionnaires. In two patients, early intervention to increase analgesia was required due to insufficient reduction in VAS score in week 2 and 3. At the end, 75% reported clinically insignificant pain (VAS score 0–1).

**Conclusion** Home-based technology-enabled follow-up for rib fractures is feasible and demonstrates potential for improving patient care through remote monitoring and pain management. Preliminary results are promising, but further research with larger samples and extended follow-up is required to confirm the long-term benefits and effectiveness.

### P27. The pathophysiology of shock following traumatic arterial injuries and the implications for resuscitation. Part 1: a hypothesis generating review of the arterial pressure reservoir

#### Carl Evans^1^, Robbie Lendrum^2^, Ewoud ter Avest^2^, Zane Perkins^2^

##### ^1^East Sussex Healthcare NHS Trust, England; ^2^London’s Air Ambulance, England

**Correspondence:** Carl Evans (carl.evans@nhs.net)

*Scandinavian Journal of Trauma, Resuscitation and Emergency Medicine* 2024, **32(S1)**: P27

**Introduction and Aims **Following injury, the body’s ability to generate an adequate blood pressure to maintain cellular perfusion is impaired. Whilst the pathophysiology of haemorrhagic shock from visceral, soft tissue and orthopaedic injury is well described; very little is known regarding the impact that arterial injury has upon cellular perfusion, aside from as a result of volume loss.

This review aims to describe the mechanisms by which blood pressure is generated to determine how arterial injury impairs cellular perfusion.

**Methods **A literature review was conducted to identify how the body generates arterial pressure and how an injury to this system has the potential to disrupt cellular perfusion.

**Result **The literature suggests that forceful left ventricular ejection determines systolic pressure, and the arterial pressure reservoir maintains pressure during diastole. The arterial pressure reservoir describes the ability of the elastic arteries to store potential energy during systole, gently recoiling during diastole to ensure continuous cellular perfusion. Of particular importance within this system is how the left ventricle is dependent upon an adequate diastolic pressure to maintain coronary perfusion [1].

**Hypothesis/Conclusion **We hypothesise that an injury to an elastic artery will impair the arterial pressure reservoir, leading to a low diastolic pressure and thus a wide pulse pressure. As a result of this, coronary perfusion will be impaired and will lead to a secondary ischaemic cardiac injury. Therefore, we suggest that the nature of arterial injury extends beyond that of volume loss, where damage to the arterial vasculature directly impairs coronary perfusion thus inhibiting cardiac compensation leading to rapid, profound shock and subsequent cardiac arrest.

**Reference**

1. Davies JE, Hadjiloizou N, Leibovich D, Malaweera A, Alastruey-Arimon J, Whinnett ZI, et al. Importance of the aortic reservoir in determining the shape of the arterial pressure waveform: the forgotten lessons of Frank. Artery Res [Internet]. 2007;1(2):40–5. Available from: https://www.sciencedirect.com/science/article/pii/S187293120700155X.

### P28. Techniques in use to identify a preventable prehospital trauma death: a systematic review

#### Carl Evans^1^, Claire Park^2^

##### ^1^East Sussex Healthcare NHS Trust; ^2^London’s Air Ambulance

**Correspondence:** Carl Evans (carl.evans@nhs.net)

*Scandinavian Journal of Trauma, Resuscitation and Emergency Medicine* 2024, **32(S1)**: P28

**Introduction and Aims** The case review process is a core component of prehospital clinical governance systems, a significant part of which is the review of prehospital fatalities to ascertain whether the death was preventable [1]. However, there is significant variation in the methodology utilised to achieve this we sought to identify and describe all published techniques used to review prehospital deaths following injury to determine preventability.

**Methods** A systematic search of Pubmed, Embase and Ovid was conducted from inception to March 2024, to identify studies which described the retrospective review of prehospital trauma fatalities and commented on whether the deaths were preventable. Studies were eligible if they presented sufficient data relating to the methodology utilised to arrive at this conclusion.

**Results **Forty-nine studies were eligible for inclusion. Fourteen described the preventability of prehospital deaths, whilst thirty-five described both prehospital and in hospital deaths. All studies but one utilised post-mortem data. The majority (36, 73%) then used a panel review to determine preventability, whereas thirteen (27%) used injury data in isolation, such as the injury severity score and probability of survival calculations with pre-determined thresholds to identify preventable deaths. Despite all patients in this review dying in the prehospital phase, prehospital professionals only appeared in fifteen out of thirty-six panels (42%). For the fourteen studies describing only prehospital deaths, no prehospital clinicians featured within the panels. The commonest outcome following review (15, 31%) was for the death to be allocated one of three classes; ‘non-preventable, potentially preventable or preventable’, however 18 variations of outcome were identified.

**Conclusion** There is significant variation in techniques used to determine preventability following fatal prehospital injuries. This is likely to be because there is no consensus on the optimal methodology, which urgently needs to be described and warrants further research.

**Reference**

1. Carenzo L, Baker C, Jones S, Hurst T. A framework for case-based learning in prehospital medicine: the London’s air ambulance experience. Air Med J [Internet]. 2022;41(6):521–5. Available from: 10.1016/j.amj.2022.09.005.

### P29. Coordinating multidisciplinary care following out-of-hospital cardiac arrest: the out-of-hospital cardiac arrest MDT meeting

#### Nikos Gorgoraptis^1^, Meadbh Keenan^2^, John Ridgway^2^, Simon Hamilton^2^, Stephen Hamshere^3^, Tom Trevarthen^2^, Anthony Bastin^4^

##### ^1^Department of Neurology, Barts Health NHS Trust, London, United Kingdom; ^2^Department of Therapies, Barts Health NHS Trust, London, United Kingdom; ^3^Department of Cardiology, Barts Health NHS Trust, London, United Kingdom; ^4^Department of Perioperative Medicine, Barts Health NHS Trust, London, United Kingdom

**Correspondence:** Nikos Gorgoraptis (nikolaos.gorgoraptis@nhs.net)

*Scandinavian Journal of Trauma, Resuscitation and Emergency Medicine* 2024, **32(S1)**: P29

Care needs following out-of-hospital cardiac arrest (OOHCA) are often complex, requiring expert input from multiple disciplines in the inpatient setting, spanning across intensive care, cardiology, neurology, neurorehabilitation and cardiac rehabilitation [1]. Effective coordination of the multidisciplinary team is necessary to achieve good care. The 2021 National Confidential Enquiry into Patient Outcome and Death (NCEPOD) rated overall care as good in only 50% of patients admitted to hospital following OOHCA and found there was room for improvement in clinical care for 36.5% and in the organisation of care for 15.6% of patients [2].

To improve care organisation, we have established a weekly inpatient OOHCA MDT at Barts Heart Centre. The MDT has input from intensive care (consultant, family liaison nurse), cardiology (interventional, cardiomyopathy, electrophysiology), neurology, occupational therapy, physiotherapy, speech and language therapy and cardiac rehabilitation. All OOHCA inpatients are discussed weekly until after discharge from Barts Heart Centre to ensure coordination of inpatient care and continuity of care post-discharge. The inpatient MDT is integrated with the Barts Heart Centre outpatient OOHCA follow-up clinic. The experience of MDT participants was examined in an online survey.

From September 2021 to August 2024, 416 individual patients [19% female, 82% male; median age at OOHCA: 60 years (range: 18-93); shockable rhythm at presentation in 74%; 62% were alive at hospital discharge or transfer, 38% died in hospital] were discussed at the weekly MDT, with a median 2 (range: 1–20) MDT discussions per patient. Participants found the MDT had a positive impact on care coordination and continuity. Representative cases are presented to illustrate how the OOHCA MDT led to better joined-up care.

Our experience suggests the OOHCA MDT may be an effective and sustainable model to achieve better coordinated care following OOHCA in specialist cardiac centres.

**References**

1. Nolan JP et al. European Resuscitation Council and European Society of Intensive Care Medicine Guidelines 2021: Post-resuscitation care’, *Resuscitation*, vol. 161, pp. 220–269, Apr. 2021, 10.1016/j.resuscitation.2021.02.012.
2. ‘NCEPOD—Out of Hospital Cardiac Arrests: (2019)’. [Online]. Available: https://www.ncepod.org.uk/2021ohca.html.

### P30. Assessment of cognitive load in clinicians performing pre-hospital REBOA: development and validation of a novel prehospital assessment tool

#### Codey Simmons^1^, Samy Sadek^2^, Rob Greenhalgh^2^, Robbie Lendrum^2^, Zane Perkins^2^, Max Marsden^3,4^

##### ^1^Barts & The London School of Medicine and Dentistry, Queen Mary University of London, London, United Kingdom; ^2^London’s Air Ambulance, The Royal London Hospital, London, United Kingdom; ^3^Centre for Trauma Sciences, Blizard Institute, Queen Mary University of London, London, United Kingdom; ^4^Academic Department of Military Surgery and Trauma, RCI, Defence Medical Services, London, United Kingdom

**Correspondence:** Codey Simmons (c.a.simmons@smd19.qmul.ac.uk)

*Scandinavian Journal of Trauma, Resuscitation and Emergency Medicine* 2024, **32(S1)**: P30

**Introduction** Pre-hospital Resuscitate Endovascular Balloon Occlusion of the Aorta (REBOA) is a time critical, complex and potentially life-saving procedure for patients with exsanguinating sub-diaphragmatic haemorrhage. The decision to perform REBOA and its technical complexities generate high levels of cognitive load. This study aimed to develop, validate and assess the feasibility of a novel pre-hospital REBOA cognitive load assessment tool.

**Method** The novel Pre-hospital REBOA Cognitive Load Assessment (PROCLASS) Tool was developed from the NASA-TLX tool after a systematic review of the literature and input from domain experts. Feasibility of assessing cognitive load with the PROCLASS tool was assessed in a simulation study during standard pre-hospital REBOA training between February to April 2024. Dual assessment of cognitive load was undertaken using objective (heart rate variability (HRV)) and subjective (PROCLASS) measurements simultaneously. Ethical permission was granted by QMUL.

**Results** The PROCLASS tool divides cognitive load into six domains: Mental Demand, Physical Demand, Temporal Demand, Performance, Situational Stress and Distractions. The domains are self-scored (0–100) and then multiplied by their relative ranking (0–5).

Five pre-hospital clinicians were enrolled, with a median duration of 5 years pre-hospital practice and 10 h of REBOA training. PROCLASS analysis highlighted similarities in the domains of cognitive load between clinicians. Mental Demand (mean = 256) and Temporal Demand (mean = 266) domains incurred the greatest burden on cognitive load, with Physical Demand (mean = 16) contributing the least. The objective assessment of cognitive load using HRV metrics showed increased sympathetic and decreased parasympathetic activation (four frequency/three time-domain variables).

**Conclusion** This study is the first to measure cognitive load in clinicians performing pre-hospital REBOA. Early findings confirm that PROCLASS is a viable tool for this purpose, providing granular data, which can be easily combined with HRV measurement. PROCLASS is currently being implemented in real-life situations with EAAA ERICA-ARREST Trial.

### P31. Requirement for emergency neurosurgery following pre-hospital or emergency department anaesthesia for suspected traumatic brain injury

#### Katie Matfin^1^, Freya Reeves-Ward^1^, Richard A Bayliss^2^

##### ^1^School of Medicine, University of Leeds, West Yorkshire, UK; ^2^Adult Critical Care, Leeds Teaching Hospitals NHS Trust, West Yorkshire, UK

**Correspondence:** Richard A Bayliss (richard.bayliss@doctors.org.uk)

*Scandinavian Journal of Trauma, Resuscitation and Emergency Medicine* 2024, **32(S1)**: P31

**Background **Delay to surgical intervention worsens outcome in patients with some traumatic brain injuries [1]. Pre-hospital emergency anaesthesia (PHEA) may cause such delay. Other patients with traumatic brain injury (TBI) do not require neurosurgery. Definitive care for these patients includes tracheal intubation and control of ventilation. The aim of this investigation was to identify the proportion of patients who were anaesthetised prior to arrival at hospital or in the emergency department who subsequently received emergent neurosurgery.

**Methods **Retrospective review was carried out of all patients for whom a trauma call was declared at Leeds General Infirmary from 1st Jan 2023 to 31st Dec 2024. Patients were included if they had a TBI and were intubated prior to arrival or in hospital. Patient demographics, injury and surgical details were recorded on a dedicated proforma.

**Results** 62 patients were included: 81% were male (n = 52); mean age was 47 years (range 18–90 years). The most common mechanisms of injury were falls (49%, n = 30) and road traffic collisions (33%, n = 20). PHEA was administered to 22 patients (35%). Neurosurgical operations (excluding intra-cranial pressure monitoring device insertion) were performed within 4 h of hospital arrival in 8 patients (13%). For patients receiving PHEA the median time from injury to definitive care was shorter (50 min, IQR 45–79 min) compared to those patients anaesthetised in the hospital (82 min, IQR 72–103 min, *p* = 0.003).

**Discussion** Emergency neurosurgical operations are infrequently required for patients following traumatic brain injury. Emergency critical care to minimise the risk of secondary brain injury will represent definitive care for most patients. This can be provided early after injury by pre-hospital enhanced care teams. Further work should investigate factors that predict the need for surgery so rapid transport to hospital and the operating theatre can be prioritised.

**Reference**

1. Poon WS, Li AKC. Comparison of management outcome of primary and secondary referred patients with traumatic extradural haematoma in a neurosurgical unit. Injury 1991; 22: 323–325.

### P32. NASA Task Load Index assessment of workload during human external cargo operations in norwegian helicopter emergency medical services

#### Håvard Mattingsdal^1^, Thomas Nordgaard Dahle^1^, Signe Søvik^2^ and William Ottestad.^1^

##### ^1^Department of Research and Development, Air Ambulance Foundation, Oslo, Norway; ^2^Department of Anaesthesia and Intensive Care, Akershus University Hospital, Lørenskog, Norway

**Correspondence:** Håvard Mattingsdal (haavard.mattingsdal@norskluftambulanse.no)

*Scandinavian Journal of Trauma, Resuscitation and Emergency Medicine* 2024, **32(S1)**: P32

**Background** Human error is a common cause in flight operation accidents. Human External Cargo operations (HEC) are complex and demand effective multi-tasking from all operators. A high workload can deplete cognitive capacity, compromising the ability to meet task demands and increasing the probability of human error [1, 2]. The Norwegian Air Ambulance Foundation is investigating the feasibility of transitioning from static Rope Rescue Operations (RRO) to Helicopter Hoist Operations (HHO). We aimed to quantify workload during rescue operations in a three-crew helicopter operation.

**Method** Study crews proficient in both methods solved multiple standardised rescue missions. Missions were divided into three phases; reconnaissance, rescuer insertion- and extraction. Overall workload was registered with the NASA Task Load Index (NASA-TLX) [3]. The study included four experienced pilots, three hoist operators and three rescuers. HEC method sequence and rescue missions were randomised. All crews solved each mission with both methods. To account for repeated measures within individuals, linear mixed-model regression analyses were performed in JMP 17.0.0 (SAS Institute, Cary, NC). *P* values < 0.05 were considered statistically significant. Values are presented as median (range).

**Results** 94 rescue missions were analysed, HHO (n = 47), RRO (n = 47). For pilots, hoist operators and rescuers, across all phases of the mission, workload was lower during HHO than RRO (all *P* < 0.003). Pilots especially experienced lower workload when performing HHO, their median workload during the rescuer insertion phase being for HHO: 37 (12–62) vs. for RRO: 52 (10–78). Compared to RRO, mixed modelling predicted the HHO method to decrease pilot workload during rescuer insertion by 13.7 points on the NASA-TLX scale (95% confidence interval 8.8–18.7, R^2^ = 0.58, *P* < 0.0001).

**Conclusion** Pilots had significantly lower perceived workload when conducting HHO as compared to RRO. This may potentially improve flight safety in HEC operations when transitioning from RRO to HHO.

**References**

1. Kharoufah H, Murray J, Baxter G, Wild G. A review of human factors causations in commercial air transport accidents and incidents. Prog Aerosp Sci 2018, 99:1–13. 10.1016/j.paerosci.2018.03.002.
2. Masi G, Amprimo G, Ferraris C, Priano L: Stress and workload assessment in aviation—anarrative review. Sensors (Basel). 2023; 23:3556. 10.3390/s23073556.
3. Hart SG: NASA-task load index (NASA-TLX); 20 years later. In Proceedings of the human factors and ergonomics society annual meeting 2006, 50:904–908. 10.1177/154193120605000909.

### P33.Cycling injuries in London: a decade-long comparative analysis of injury patterns

#### Yi Lun Khaw^1^, Georgia Lekka^1^, Imogen K Shaw^1^, Tristram J D Reames^1^, Anne Weaver^2^, Charlotte Lindsay^2^, Ross Davenport^2^

##### ^1^Barts and the London, Queen Mary University of London, London, UK; ^2^Barts Health NHS Trust, London, UK

**Correspondence:** Ross Davenport (ross.davenport@qmul.ac.uk.)

*Scandinavian Journal of Trauma, Resuscitation and Emergency Medicine* 2024, **32(S1)**: P33

**Background** Each year 16,000 cyclists are killed or injured on UK roads but little is known about injury patterns [1]. The protective effects of helmet use are well described but potential impacts of novel devices to protect other body regions is unknown [2]. The aim of this service evaluation was to characterise anatomical injury patterns to inform injury prevention efforts for cyclists.

**Methods** All cyclists admitted to the Royal London Major Trauma Centre (January 2012—December 2021) with documented Injury Severity Scores (ISS) were included. Severe injury was defined as Abbreviated Injury Scale (AIS ≥ 3) in at least one body region.

**Results** 823 cyclists were analysed: median age 35 years, 17% female, 3% in-hospital mortality. Half of injuries occurred following collisions with small vehicles (car/motorcycle/bicycle), one third were falls from the bicycle and 10% were collisions with stationary vehicles or street furniture. Severe injuries were present in 374 (45%) patients. 80% of injuries were isolated to one body region with head (42%), lower extremity (22%) and thorax (18%) the most common.

Multiple severe injuries occurred in 74 (20%) patients of which 61% had a severe head injury. In polytrauma patients, the commonest mechanism of injury was collision with small vehicles (46%), falls (23%) and collisions with large vehicles (22%). In severe head injury patients, co-existing thoracic injuries included rib fractures (21%), pulmonary contusions/lacerations (20%), and haemothorax or pneumothorax (18%). Similar patterns were observed in patients with severe spine (n = 12) or abdominal injuries (n = 12). In patients with severe chest injuries (n = 60), head injuries (43%) were the most common co-existing injury.

**Conclusion **Severe injuries showed distinct patterns; head injuries were the most common isolated severe injury, while chest injuries frequently accompanied other severe injuries. Thoracic protection for cyclists in the form of inflatable devices may have a role in reducing severe and disabling injuries.

**References**

1. Reported road casualties in Great Britain: pedal cycle factsheet, 2022 [Internet]. GOV.UK. Available from: https://www.gov.uk/government/statistics/reported-road-casualties-great-britain-pedal-cyclist-factsheet-2022/reported-road-casualties-in-great-britain-pedal-cycle-factsheet-2022.
2. Olivier J, Creighton P. Bicycle injuries and helmet use: a systematic review and meta-analysis. International Journal of Epidemiology [Internet]. 2016 Jul 22;46(1):dyw153. Available from: https://academic.oup.com/ije/article/46/1/278/2617198.

### P34. Epidemiology of prehospital traumatic cardiac arrest in a dense urban area, a retrospective cohort study in Geneva, Switzerland

#### R. Midez^1^, L. Suppan^1^, C. Egger^2^, E. Jourdan^1^, H. Quintard^3^, T. de Valence^1^

##### ^1^Division of Emergency Medicine, Department of Anaesthesiology, Pharmacology, Intensive Care and Emergency Medicine, Geneva University Hospitals, Geneva, Switzerland; ^2^Unit of Forensic Medicine, University Center of Legal Medicine Lausanne-Geneva (CURML), Geneva University Hospitals and University of Geneva, Geneva, Switzerland; ^3^Division of Intensive Care Medicine, Department of Anaesthesiology, Pharmacology, Intensive Care and Emergency Medicine, Geneva University Hospitals, Geneva, Switzerland

**Correspondence:** R. Midez (Remy.midez@etu.unige.ch)

*Scandinavian Journal of Trauma, Resuscitation and Emergency Medicine* 2024, **32(S1)**: P34

**Background** Traumatic cardiac arrest (TCA) has previously been viewed as a non-survivable event [1]. Advances in prehospital resuscitation have improved TCA survival, and current guidelines emphasize addressing reversible causes [2]. Understanding local data on the characteristics and outcomes of TCA may help in improving prevention and care.

**Method** We conducted a retrospective cohort study of all patients who experienced a prehospital TCA in Geneva from January 1st, 2008 to December 31st, 2022. Data was extracted from the databases of the prehospital physician-led emergency service (SMUR) and the coroner’s office. This trial was approved by the regional research ethics committee (Project-ID: 2023-00767).

**Results** During the 15-year period, there were 673 cases of TCA. 464 (69%) were managed by the SMUR. The mean (standard deviation) patient age was 51.8 (21.6) and 485 (72.1%) were male. Of the patients treated by the SMUR, 341 (73.5%) sustained blunt trauma, 92 (19.8%) had penetrating trauma, and 19 (4.1%) suffered burns. The most common mechanisms of injury were falls from height (39.2%), road traffic accidents (24.2%), and firearms injuries (14.9%). Head injuries were observed in 62% of patients and 41.2% experienced hemorrhage. Initial rhythm was asystole in 198 (42.7%) cases, and pulseless electrical activity in 115 (24.8%). Most patients (90.5%) went into cardiac arrest before the SMUR’s arrival. Emergency response time was 8.4 (4.4) minutes, on-scene time was 34.7 (18.9) minutes and transport time was 6.4 (4.3) minutes. In patients who underwent resuscitation, 167 (79.9%) had an advanced airway inserted, 65 (31.1%) had needle or finger chest decompression, 146 (69.9%) received adrenaline, and 168 (80.4%) received fluids. Survival to hospital admission was 13% (8%).

**Conclusion** In Geneva, TCAs occur more than 44 times yearly with no indication of decline. This data will help to suggest and implement interventions aimed at improving outcomes, tailored to the needs of this specific patient population.

**References**

1. Rosemurgy AS, Norris PA, Olson SM, Hurst JM, Albrink MH. Prehospital traumatic cardiac arrest: the cost of futility. J Trauma. 1993 Sep;35(3):468–73; discussion 473–474.
2. Lott C, Truhlář A, Alfonzo A, Barelli A, González-Salvado V, Hinkelbein J, et al. European Resuscitation Council Guidelines 2021: Cardiac arrest in special circumstances. Resuscitation. 2021 Apr;161:152–219.

### P35. Multi-lumen catheter insertion during trauma resuscitation: assessing clinical outcomes and complications

#### Jinjoo Kim^1^, Sora Kim^2^, Jayoung Yoo^2^, Jonghwan Moon^1^

##### ^1^Division of Trauma Surgery, Department of Surgery, Ajou University School of Medicine, Suwon, Republic of Korea; ^2^Ajou University hospital, Suwon, Republic of Korea. 

**Correspondence:** Jonghwan Moon (soyo1226@naver.com)

*Scandinavian Journal of Trauma, Resuscitation and Emergency Medicine* 2024, **32(S1)**: P35

**Background** Intravascular access in polytrauma patients with haemorrhagic shock is challenging.[1] Therefore, the placement of a 9-Fr Multi-lumen Access Catheter (MAC, Arrow,Teleflex, PA, USA) is frequently required during trauma resuscitation; however, little evidence exists on the optimal site for insertion.[2] This study aims to evaluate the impact of MAC insertion site on mortality and complication rate in blunt trauma patients.

**Materials and Methods **This study was conducted retrospectively at a single level-one trauma centre. It included blunt trauma patients (ISS ≥ 15) aged ≥ 18 years who needed MAC placement in the trauma bays during resuscitation between January 2019 and December 2022. All patients underwent massive transfusions. Mortality and procedural complications according to the catheter insertion site, including femoral vein (FV) and subclavian vein (SCV), were collected.

**Results** In total 382 patients were included in analysis. Of these patients, 212 (55.5%) were finally inserted with SCV catheter, and 170 (44.5%) had FV catheters. There was no significant differences in Injury Severity Score (ISS) (37.6 ± 11.1 in SCV group vs. 37.2 ± 13.3 in FV group, *p* = *0.753*), 7-day mortality (7.1% in SCV group vs. 10.6% in FV group, *p* = *0.225*) and amount of trausfused red blood cells (10.1 ± 5.9units vs. 10.3 ± 6.5units, *p* = *0.777*) and fresh frozen plasma (9.1 ± 5.8units vs. 9.6 ± 6.3units, *p* = *0.435*). Total 402 MAC insertions were performed for 382 patients. 230 SCV catheterizations and 172 FV catheterizations were attemped. Failed catheterization was more common in SCV (8.7% vs. 1.2%, *p* = *0.001*), and haemorrhage requiring treatment occured only in SCV group (0.9% vs. 0%, *p* < *0.001*). Although 19.2% of FV catheterisations developed deep vein thromboses, pulmonary thromboembolism did not develop.

**Conclusion** During trauma resuscitation, the insertion site of MAC does not affect clinical outcome. However, considering complications related to the insertion site, it is necessary to make a decision for optimal insertion site based on the patient’s condition and physicians’ level of proficiency.

**References**

1. Dumas RP, Vella MA, Maiga AW, et al. Moving the needle on time to resuscitation: An EAST prospective multicenter study of vascular access in hypotensive injured patients using trauma video review. J Trauma Acute Care Surg. 2023; 95(1):87–93. 10.1097/TA.0000000000003958.
2. David V. Feliciano, et al. Trauma, 9e Eds. McGraw Hill; 2020.

### P36. Red cell transfusion and hyperkalaemia in trauma patients: a single centre experience

#### Rachel Tresman^1^, Quang Nguyen^1^, Julie Cole^1^, Katherine Hunter^1^, C Mark Harper^1^, Stephanie Tilston^1^

##### ^1^University Hospitals Sussex NHS Foundation Trust

**Correspondence:** Quang Nguyen (quang.nguyen1@nhs.net)

*Scandinavian Journal of Trauma, Resuscitation and Emergency Medicine* 2024, **32(S1)**: P36

**Background **Despite a historical association between blood transfusion and hyperkalaemia risk, this has not been convincingly borne out in clinical studies [1]. The data on this relationship in trauma patients is very limited and similarly inconclusive [2, 3], despite it being potentially significant given their propensity for haemorrhage, shock, and acidosis. We sought to analyse data from a major trauma centre to investigate the relationship between emergency red cell transfusion requirements and the incidence of hyperkalaemia in our trauma population.

**Methods **Data were provided by the hospital transfusion department as to which patients had received non-crossmatched (emergency) blood between January 2022 and December 2023. From these, trauma patients requiring blood within the first 24 h of admission were selected for analysis. The number of red cell units transfused and the peak plasma potassium level from blood gas results in the first 24 h was recorded. Hyperkalaemia was defined as mild 5.5–5.9 mmol/L, moderate 6–6.4 mmol/L, and severe > 6.5 mmol/L.

**Results** Data were analysed from 131 patients. Linear regression analysis demonstrated a significant association between number of red cell units transfused and peak potassium level (*p* < 0.0001). Incidence of hyperkalaemia in patients receiving 1–4 units of red cells (n = 88) was 30.7% and severe hyperkalaemia 11.4%. Incidence in patients receiving 5–8 units (n = 33) was 43.5% and severe was 25%. In > 8 units (n = 10) incidence of hyperkalaemia and severe hyperkalaemia was 50%.

**Conclusions **A statistically significant correlation between number of red cell units transfused and incidence of hyperkalaemia was found in our trauma cohort. Further data is needed for patients requiring > 8 units, and to identify any risk factors for hyperkalaemia. There remains an absence of evidence and guidelines regarding management of transfusion-related hyperkalaemia in trauma patients, and we believe that these data should prompt consideration for inclusion of such guidelines in trauma protocols.

**References**

1. Wolf J, Geneen LJ, Meli A, Doree C, Cardigan R, New HV. Hyperkalaemia following blood transfusion-a systematic review assessing evidence and risks. Transfus Med Rev. 2022;36(3):133–142.
2. Aboudara MC, Hurst FP, Abbott KC, Perkins RM. Hyperkalemia after packed red blood cell transfusion in trauma patients. J Trauma. 2008;64(2 Suppl):S86-S91.
3. Au BK, Dutton WD, Zaydfudim V, Nunez TC, Young PP, Cotton BA. Hyperkalemia following massive transfusion in trauma. J Surg Res. 2009;157(2):2

### P37. Where can advanced prehospital services add the most value in preventable trauma deaths?

#### Aditi Nijhawan^1^, Ewoud ter Avest^1^, Callum J Twohig^2^, Flora Bird^1^, Virginia Fitzpatrick-Swallow^3^, Robbie Lendrum^1^, David J Lockey^1^, Zane B Perkins^1^

##### ^1^London’s Air Ambulance, London, UK; ^2^Centre for Trauma Sciences, Queen Mary University of London. London, UK; ^3^Home Office Registered Forensic Pathologist, Wantage, United Kingdom

**Correspondence:** Aditi Nijhawan (aditi.nijhawan1@nhs.net)

*Scandinavian Journal of Trauma, Resuscitation and Emergency Medicine* 2024, **32(S1)**: P37

**Background **Many patients who die after trauma do so early after their injury [1, 2]. As prehospital deaths are not included in national trauma registries and strict governance exists around post-mortem reports, exactly when and why these patients die remains unknown. Filling these gaps could help guide prehospital services in establishing targeted interventions for their populations.

**Methods **In a retrospective cohort study, all adult trauma patients attended by London’s Air Ambulance who died prehospital or in-hospital between January 2019 and December 2020 were analysed to establish their time and cause of death. Likely cause of death was established through assessment of surgical, radiological and post-mortem findings.

**Results **During the study period, LAA treated 3089 adult trauma patients, of which 497 (16.1%) died. Of all trauma deaths, 383 (77.1%) had a prehospital traumatic cardiac arrest. Most deaths (80.1%) occurred within a day of injury, with a median time from injury to prehospital traumatic cardiac arrest (TCA) of 12 min (IQR: 6–24). Likely cause of death was determined for 97.0% of patients. The leading causes of death were Central Nervous System (CNS) injuries (39.0%) followed by haemorrhage (36.8%). All deaths from hypoxia, tension pneumothorax and cardiac tamponade occurred prior to hospital admission. TCA from haemorrhage was predominantly prehospital, with 96.2% occurring before admission. In-hospital deaths were predominantly due to CNS injury (84.2%), followed by Multi-Organ Dysfunction Syndrome (7.0%), haemorrhage (6.1%), and in-hospital complications (2.6%).

**Discussion **These findings show that the largest proportion of potentially survivable trauma related deaths occur prehospitally. Given the narrow time window from injury to arrest, provision of advanced interventions soon after injury could lead to more survivors. Trauma systems must employ thorough data collection of prehospital trauma fatalities to better understand and manage this group of patients.

**References**

1. Holcomb JB. Transport time and preoperating room hemostatic interventions are important: improving outcomes after severe truncal injury. Crit Care Med. 2018;46(3):447–453.
2. Carroll SL, Dye DW, Smedley WA, et al. Early and prehospital trauma deaths: who might benefit from advanced resuscitative care? J Trauma Acute Care Surg. 2020;88(6):776–782.

### P38. Insights into the management of code red trauma at a London Major Trauma Centre: a one year retrospective study

#### Edmund Lodwick^1^, Cosmo Scurr^2^

##### ^1^Emergency Medicine Department, Chelsea and Westminster Healthcare NHS Trust, London, UK; ^2^ Anaesthesia Department, St. Mary’s Hospital, Imperial College Healthcare NHS Trust, London, UK

**Correspondence:** Edmund Lodwick (Edmund.lodwick@nhs.net)

*Scandinavian Journal of Trauma, Resuscitation and Emergency Medicine* 2024, **32(S1)**: P38

**Background **Haemorrhage accounts for approximately 40% of deaths in patients suffering from major traumatic injury [1]. A significant proportion of trauma admissions to St Mary’s Hospital, a London Major Trauma Centre, are identified to have suffered major haemorrhage and are declared a Code Red trauma. The aim of our study was to determine the profile of these patients and assess factors show to impact morbidity and mortality, including transfusion ratios, time to damage control surgery (DCS) initiation and use of thromboelatography.

**Method **This retrospective service evaluation reviewed adult (> 16 years old) patients with traumatic major haemorrhage admitted to a mature Major Trauma Centre (MTC) as Code Red trauma, from 1st January 2022 to 31st December 2022. Data was compiled from electronic patients records, transfusion laboratory records and scanned paper trauma booklets.

**Results **Of 87 patients were identified nearly 90% were male, with > 50% less that 35 years old. Stabbings and road traffic accidents (RTAs) accounted for > 50% of cases. 86% suffered polytrauma. Major haemorrhage was identified by pre-hospital clinicians in 72% of patients. Average time from hospital arrival to theatre start was 36 min in those taken for DCS. Overall inpatient mortality was 12.6%, all due to blunt injuries. Transfusion ratios were close to national guidelines. 14% required massive transfusion, associated with 20% mortality. Thromboelastography was used in 80% of cases going for DCS. 3 patients underwent resuscitative thoracotomy, of which 2 survived to discharge.

**Conclusion **Patients suffering traumatic major haemorrhage tend to be young men. RTAs resulted in greatest mortality. Overall mortality has decreased compared to 2021–2022. If requiring DCS, transfer to theatre is swift. Balanced transfusion is achieved in the majority of cases, however the impact of increasing pre-hospital transfusion requires further investigation. Thromboelastography use is variable depending on patient disposition and destination.

**Reference**

1. Curry NS, Davenport R. Transfusion strategies for major haemorrhage in trauma. Br J Haematol. 2019;184: 508–23.

### P39. Haemodynamic effects of thigh cuff inflation during simulated haemorrhage in healthy volunteers

#### Sara Stadskleiv Torbjørnsen^1^, Sole Lindvåg Lie ^2^, Jonny Hisdal^1^, Marius Rehn^2^, Lars Øivind Høiseth.^3^

##### ^1^Institute of Clinical Medicine, Faculty of Medicine, University of Oslo, Oslo, Norway; ^2^Department of Research and Development, Norwegian Air Ambulance Foundation, Oslo, Norway; ^3^Department of Anaesthesia and Intensive Care Medicine, Division of Emergencies and Critical Care, Oslo University Hospital, Oslo, Norway

**Correspondence:** Sara Stadskleiv Torbjørnsen (sarastadskleiv@gmail.com)

*Scandinavian Journal of Trauma, Resuscitation and Emergency Medicine* 2024, **32(S1)**: P39

**Background** Resuscitative endovascular balloon occlusion of the aorta (REBOA) aims to control noncompressible haemorrhage in trauma patients. REBOA has mainly been studied in animals and trauma patients. We aim to study whether thigh cuff inflation during simulated haemorrhage can serve as an experimental human model of zone 3 REBOA.

**Methods** Twenty healthy volunteers will be exposed to suprasystolic bilateral proximal thigh cuff inflation to mimic the haemodynamic effects of REBOA during simulated haemorrhage using lower body negative pressure (LBNP) (1,2). Each volunteer will undergo two experimental conditions in a randomised order during the same visit. In one condition, volunteers will be exposed to 60 mmHg of LBNP for six minutes. In the other condition, 60 mmHg of LBNP will be applied for six minutes, in addition to thigh cuff inflation during the last three minutes. Middle cerebral artery blood velocity and cerebral oxygenation will be measured as indicators of cerebral haemodynamics. Systemic haemodynamic parameters, including blood pressure, stroke volume and heart rate, will be measured continuously and non-invasively. The effect of thigh cuff inflation on systemic and cerebral haemodynamics will be assessed using mixed-effects regression analyses. Pain associated with thigh cuff inflation will be assessed using a numerical rating scale (NRS).

**Results** The study is currently enrolling volunteers. Preliminary systemic haemodynamic results from one volunteer indicate no changes in SV, HR, or CO during thigh cuff inflation, while MAP increased by 13 mmHg. Thigh cuff inflation was associated with a NRS of 4.0.

**Conclusion** Combining LBNP and thigh cuffs is feasible, although pain may confound the haemodynamic effects of thigh cuff inflation. Evaluation of data from all volunteers is necessary to conclude on the effects of thigh cuff inflation on cerebral and systemic haemodynamics during simulated haemorrhage.

**References**

1. Goswami N, Blaber AP, Hinghofer-Szalkay H, Convertino VA. Lower body negative pressure: physiological effects, applications, and implementation. Physiol Rev. 2019 Jan;99(1):807–51.
2. Lie SL, Hisdal J, Rehn M, Høiseth LØ. Effect of systemic vascular resistance on the agreement between stroke volume by non-invasive pulse wave analysis and Doppler ultrasound in healthy volunteers. PLOS ONE. 2024 May 7;19(5):e0302159.

### P40. Transfer to cardiac arrest centre increases survival from out of hospital cardiac arrest

#### Terry P Brown^1^, Joyce Yeung^2,3^, Christopher Smith^2^, Adam J Boulton^2^

##### ^1^Applied Research Collaboration West Midlands, Warwick Medical School, University of Warwick, Coventry, UK; ^2^Warwick Medical School, University of Warwick, Coventry, UK; ^3^Critical Care Unit, University Hospitals Birmingham NHS Foundation Trust, Birmingham, UK

**Correspondnce:** Terry P Brown (t.brown.1@warwick.ac.uk)

*Scandinavian Journal of Trauma, Resuscitation and Emergency Medicine* 2024, **32(S1)**: P40

**Background** In UK there are over 100,000 calls to ambulance services to attend an out-of-hospital cardiac arrest (OHCA), and over 40,000 where resuscitation was started or continued after their arrival on scene. The International Liaison Committee on Resuscitation suggest that patients with OHCA should be transported to cardiac arrest centres (CACs), although the evidence was of very low certainty but significant knowledge gaps remain [1]. Recently two UK studies have found conflicting results: one reporting no reduction in deaths in patients transferred to a CAC [2]; the other reporting a 44% increase in survival to hospital discharge [3]. The aim of the current analysis was to examine the survival of OHCA patients in England directly transferred to a CAC.

**Methods **Data held by the OHCA Outcomes registry, hosted by the University of Warwick, on OHCAs occurring between 2018 and 2023 were analysed. The distance between the OHCA location and a) the hospital to which the patient was transferred to and b) the nearest CAC were calculated. The primary outcome was survival (to hospital discharge or 30-days).

**Results **A total of 180,798 non-traumatic cases were reviewed. Of these, 72,871 were transported to hospital, with 32,628 (44.8%) directly to a CAC. Of those transported to a non-CAC (n = 40,243) a CAC was nearer in 31.6%. In all cases, the odds of survival in patients transferred directly to a CAC was significantly greater than in those taken elsewhere (OR = 1.26, 95% CI 1.21–1.31). Survival benefit remained when comparing shockable/non-shockable rhythm, Utstein comparator group, male/female, age groups, urban/rural.

**Conclusion **We have shown in a retrospective observational analysis of a large number of cases that direct transfer to a designated CAC of a patient sustaining an OHCA significantly increases the chances of survival. This increases the evidence that such patients should, where possible, be cared for at such centres of excellence.

**Acknowledgements**

Members of the Out-of-Hospital Cardiac Arrest Outcomes registry and English Ambulance Services.

**References**

1. Boulton AJ et al. Cardiac arrest centres for patients with non-traumatic cardiac arrest: a systematic review. Resuscitation, 2024;110–387.
2. Patterson T et al. Expedited transfer to a cardiac arrest centre for non-ST-elevation out-of-hospital cardiac arrest (ARREST): a UK prospective, multicentre, parallel, randomised clinical trial. Lancet, 2023;402(10,410):1329–1337.
3. Price J et al. Increased survival for resuscitated Utstein-comparator group patients conveyed directly to cardiac arrest centres in a large rural and suburban population in England. Resuscitation. 2024;201:110–280.

### P41. Correlation of injuries in road traffic collisions

#### Emily R Ashworth^1^, Shehan Hettiaratchy^1^

##### ^1^Trauma@IC, Imperial College Healthcare NHS Trust, London, United Kingdom.

*Scandinavian Journal of Trauma, Resuscitation and Emergency Medicine* 2024, **32(S1)**: P41

**Background **In 2022 there were 23,465 reported collisions in London, resulting in 102 fatalities, 3859 serious injuries, and 23,246 mild injuries according to a Transport for London (TfL) report.

There is vast data on the biomechanics of individual injuries as a result of road traffic collisions, however mechanisms of injury have not been previously been explored concurrently to look at whole body injury.

As a major trauma centre, the Trauma@IC team at St Mary’s decided to explore this further using retrospective data.

**Method** All St Mary’s Hospital trauma admissions over one-year period (March 2023–March 2024) were used and filtered for all patients with road traffic collision as a mechanism of injury. In addition, AIS and exact road traffic collision mechanism was collected. Number of patients with head and neck, spine, thoracic, abdominal, pelvic, upper limb, lower limb and max fax injuries were recorded. These were then recorded, by percentage of patients with that injury on body maps. Chi-Squared and Fisher’s exact were used to look at injury constellations.

**Results** Results were divided by road traffic collision mechanism: e-scooter, cyclist, pedestrian, motorcycle and in-vehicle. There were 503 Road traffic collisions in a one-year period in North West London. Lower limb injury was the most common injury with 31% of patients suffering some kind of trauma. This was followed by chest and head injuries which were 19% and 18% of patients respectively. The body maps showed that motorcyclists and e-scooter rider were most likely to suffer lower limb injuries. There was a similar pattern of injuries between pedestrians and cyclists.

**Discussion** The data shows there are different mechanisms of injury depending on the type of transport involved in a road traffic collision. E-scooters had the most regions of the body injured. Integrating this data with outcome and Injury Severity Score would give a greater insight into the patterns and correlations of injury.

**Reference**

National Statists Road Traffic Statistics https://www.gov.uk/government/statisticaldata-sets/road-traffic-statistics-tra.

### P42. Does prehospital critical care provided by Helicopter Emergency Medical Services (HEMS) improve the outcomes of patients with traumatic brain injury? A systematic review of the literature.

#### Sarah McLachlan^1,2^, Hilary Bungay^1^, Matthew Williams^3^, Festus Meshe^1^

##### ^1^School of Allied Health & Social Care, Anglia Ruskin University, Chelmsford, Essex, UK; ^2^Essex & Herts Air Ambulance, Earls Colne, Colchester, Essex, UK. ^3^London North West University Healthcare NHS Trust, London, UK

**Correspondence:** Matthew Williams (matthew.williams39@nhs.net)

*Scandinavian Journal of Trauma, Resuscitation and Emergency Medicine* 2024, **32(S1)**: P42

**Background** Traumatic brain injury (TBI) is a prevalent and serious pathology requiring specialist and time-critical interventions [1]. Helicopter Emergency Medical Services (HEMS) deliver advanced prehospital interventions not provided by ground emergency medical services (GEMS), but a recent systematic review suggested that evidence on the benefits of HEMS on patient outcomes is equivocal [2]. However, enhanced care provided by HEMS (e.g. prehospital anaesthesia) may confer benefit in specific populations such as TBI patients. This systematic review aims to collate and appraise evidence on outcomes following TBI between HEMS and GEMS.

**Methods** The review was conducted according to the Preferred Reporting Items for Systematic Reviews and Meta-Analyses (PRIMSA) [3]. Five electronic databases were searched from inception to July 2023. Search terms were broad to capture all literature comparing patient outcomes between HEMS and GEMS; this helped to ensure that studies reporting sub-group analysis for TBI were included. The primary outcome was mortality/survival. Initial title and abstract screening was performed by a single reviewer; full text screening was undertaken by two independent reviewers. A subsequent decision was made to include only literature published from 2000.

**Results** Searches yielded a total of 7737 records. Following duplicate removal and title and abstract screening, 290 full texts were retrieved. After full text screening, 15 studies were included. Ten studies reported a mortality or survival benefit for patients attended by HEMS. In some studies, benefit was seen primarily in patients with higher injury severity. Where a survival benefit was identified, the underlying mechanisms were unclear. Other favourable outcomes of HEMS attendance were identified, for example lower rate of neurological disability.

**Discussion** There is some evidence for a mortality/survival benefit in TBI patients attended by HEMS, although included studies were largely retrospective with associated methodological limitations. Prospective studies and trials are needed to improve the quality of evidence.

**References**

1. Pelieu I, Kull C, Walder B. Prehospital and emergency care in adult patients with acute traumatic brain injury. Med. Sci. 2019;7(1):12.
2. Risgaard B, Draegert C, Baekgaard JS, Steinmetz J, Rasmussen LS. Impact of physician‐staffed helicopters on pre‐hospital patient outcomes: a systematic review. Acta Anaesthesiol Scand. 2020; 64(5):691–704.
3. Page MJ, McKenzie JE, Bossuyt PM, Boutron I, Hoffmann TC, Mulrow CD, et al. The PRISMA 2020 statement: an updated guideline for reporting systematic reviews. BMJ. 2021;372:n71.

### P43. A retrospective cohort study of quality of CT chest reporting and association with delivering of regional anaesthesia for traumatic rib fractures

#### Salman Naeem^1^, Luke Peacock^1^, Syme Bhopal^1^, Grace Catchpole^1^, Ammar Habbal^1^, Jame Bearman^1^, Ahmad Hassan^1^, Drew Gordon^1^, Simrik Sunuwar^1^, Adraina Gonzalez^1^, Hafsa Ali^1^, Mohammed Dafalla^1^, Mahika Kamat^1^, Mehdin Shah^1^, Anna Vondy^2^, Samuel Harding^3^, Hassan Ahmed^4^ Serena Rovida^5^

##### ^1^East Kent Hospitals University Foundation Trust, Ashford, United Kingdom; ^2^Warrington and Halton Teaching Hospitals NHS Foundation Trust, Warrington, United Kingdom; ^3^Royal Melbourne Hospital, Melbourne. Australia; ^4^The University of Manchester, United Kingdom; ^5^Toronto General Hospital, Canada

**Correspondence:** Luke Peacock (Luke.peacock@nhs.net)

*Scandinavian Journal of Trauma, Resuscitation and Emergency Medicine* 2024, **32(S1)**: P43

**Background** Pain control is paramount in thoracic trauma patients. Multimodal analgesia including regional anaesthesia (RA) have been used which requires reporting of exact anatomical site of ribs fractured. However, there is no guidance on reporting standards for rib fractures in UK. This retrospective study aims to investigate reporting of rib fractures on CT chest and relationship with RA.

**Methods **A retrospective cohort study in a district general hospital was conducted from January 2021 to December 2023. All patients diagnosed with rib fractures were included. Primary outcome was reporting of site and number of rib fractures. Secondary outcome was association of RA with reporting of anatomical location of rib fractures on CT reporting. Percentages were calculated for nominal data, odds ratio was used to find association of RA with CT reporting standards.

**Results** A total of 848 patients were screened and 609 included in final analysis. The population was predominantly male (60%) with mean age of 69 (SD17.2) years. Fall form standing was the most common mechanism of injury (40%) with 80% having computed tomographic scan of chest. Out of those 317 (52%) were reported by in-house radiologists, 509 (83%) had site of rib fractures reported, 574 (94%) had number of rib fractures reported and 484 (79%) had both reported. Median ribs fractured were 3 (IQR 2–4) and 122 (20%) received RA. The odds of having RA increased if both site and number of rib fractures were reported (1.14 95% CI 0.7–1.9).

**Conclusion **Reporting of anatomical location of rib fractures on CT reporting may be associated with increase in administration of RA in patients. This might help direct choice of RA being administered.

### P44. A retrospective cohort study investigating feasibility and impact of delivery of out-of-theatres regional anaesthesia for traumatic rib fractures

#### Salman Naeem^1^, Luke Peacock^1^, *Syme Bhopal^1^, Grace Catchpole^1^, Ammar Habbal^1^, James Bearman^1^, Ahmad Hassan^1^, Drew Gordon^1^, Simrik Sunuwar^1^, Adraina Gonzalez^1^, Hafsa Ali^1^, Mohammed Dafalla^1^, Mahika Kamat^1^, Mehdin Shah^1^, Anna Vondy^2^, Samuel Harding^3^, Serena Rovida^4^

##### ^1^East Kent Hospitals University Foundation Trust, Ashford, United Kingdom; ^2^Warrington and Halton Teaching Hospitals NHS Foundation Trust, Warrington, United Kingdom; ^3^Royal Melbourne Hospital, Melbourne. Australia; ^4^Toronto General Hospital, Toronto

**Correspondence:** Syme Bhopal (S.bhopal1@nhs.net)

*Scandinavian Journal of Trauma, Resuscitation and Emergency Medicine* 2024, **32(S1)**: P44

**Background **Thoracic injury accounts for 25% deaths in trauma patients. Pain control is paramount and different analgesic techniques including regional anaesthesia (RA) have been used but none has been proven to be superior in limited literature available [1]. This retrospective cohort study aims to study feasibility of delivery of RA for patients with trauma thoracic injuries and impact on mortality.

**Methods **A retrospective cohort study in a district general hospital was conducted from January 2021 to December 2023. All patients diagnosed with rib fractures were included. Primary outcome was in-hospital mortality. Secondary outcome was length of stay (LOS). Chi-square t-test and odds ratio were used to find association of mortality and independent Mann Whitney-U test was used to report difference in length of stay between two groups.

**Results **A total of 848 patients were screened and 629 included in final analysis. The population was predominantly male (60%) with mean age of 69 (SD17.2) years. Fall form standing was the most common mechanism of injury (40%) with 80% having computed tomographic scan as an initial radiological investigation. The median ribs fractured were 3 (IQR 2–4), 20% received RA for analgesia and 10% patient met the primary outcome. Both groups had similar distribution of variables. The adjusted odds of mortality decreased with RA (0.6 95% CI 0.3–1.3) and LOS was significantly lower in the intervention cohort by 1.7 days (95% CI 0.6 -4). Erector spinae plane block was the commonest RA technique (66%), 17% blocks being performed by emergency physicians, 27% of blocks had complications and catheter dislodgement was the most common complication (78%).

**Conclusion** This study highlights that RA may lower the odds of mortality in patients admitted with rib fractures. Erector spinae plane block is commonly performed block which can be performed by emergency physicians with low rates of complications.

**Reference**

1. El-Boghdadly K, Wiles MD. Regional anaesthesia for rib fractures: too many choices, too little evidence. Anaesthesia. 2019;74(5):564–568. 10.1111/anae.14634.

### P45. Effect of time to computed tomography scan on patient outcome in severely injured trauma patients

#### Pamela J. M. Tay^1^, Goh E. Shaun^2^, Sang Do Shin^3^, Kentaro Kajino^4^, Ivan S. Y. Chua^1^, for the PATOS Clinical Research Network

##### ^1^Department of Emergency Medicine, Singapore General Hospital, Singapore; ^2^Department of Emergency Medicine, Woodlands Health Campus, Singapore; ^3^Department of Emergency Medicine, Seoul National University Hospital, Seoul, Korea; ^4^Department of Emergency and Critical Care Medicine, Kansai Medical University, Hirakata, Osaka, Japan.

*Scandinavian Journal of Trauma, Resuscitation and Emergency Medicine* 2024, **32(S1)**: P45

**Background **Trauma is a leading cause of death in adults, presenting a huge financial burden to society [1, 2]. Advances in trauma resuscitation have led to reduced morbidity and mortality. Use of computed tomography (CT) imaging is now standard of care in trauma patients presenting to the emergency room and allows for rapid and accurate diagnosis of injuries sustained. We aimed to evaluate whether time to CT scan is associated with improved Modified Rankin Scale (MRS) and Glasgow Outcome Scale (GOS) in severely injured trauma patients.

**Materials and Methods **This was a retrospective cohort study of patients registered in the Pan-Asian trauma outcomes study (PATOS) [3] with injury severity score (ISS) of 15 or more. Data was obtained from PATOS, an international and multicenter trauma registry from January 2015 to December 2022. Exclusion criteria included: patients < 16 years old, ISS < 15, disposition dead or unknown, or missing data fields. Patients were allocated into two groups—those with CT scan less than 1 h of arrival in the emergency department (ED), and those with a CT scan > 1 h after arrival in the ED. Odds ratio (OR) for MRS and GOS on discharge were obtained via binary logistic regression.

**Results **4866 patients were included in the analysis (3853 with duration to CT scan less than 1 h; 1213 with CT scan > 1 h). In patients with CT scan less than 1 h, OR of good MRS was 1.41 (95% CI 1.23–1.60) and OR for GOS on discharge was 1.20 (95% CI 1.044–1.371).

**Conclusion** Shorter duration to CT scan was associated with higher OR of better MRS and GOS on discharge in major trauma patients. Processes targeted at reducing time to CT scan may further improve outcomes in this population.

Acknowledgements

The authors would like to thank the investigators and researchers from participating PATOS sites for the collaboration. PATOS Clinical Research Network: Ki Jeong Hong (South Korea), Shah Jahan Mohd Yussof (Malaysia), Khalifa Alqaydi (United Arab Emirates), Le Bao Huy (Vietnam), Bernadett Velasco (Philippines), Jen Tang Sun (Taiwan), Jirayu Chantanakomes (Thailand) and T.V. Ramakrishnan (India).

**References**

1. Colin D. Mathers, Ties Boerma, Doris Ma Fat, Global and regional causes of death, Br Med Bull. 2009;92(1):7–32, 10.1093/bmb/ldp028.
2. Choi J, Carlos G, Nassar AK, Knowlton LM, Spain DA. The impact of trauma systems on patient outcomes. Curr Probl Surg. 2021 Jan;58(1):100,849. 10.1016/j.cpsurg.2020.100849. Epub 2020 Jun 10. PMID: 33,431,134; PMCID: PMC7286246.
3. Kong SY, Shin SD, Tanaka H, Kimura A, Song KJ, Shaun GE, Holmes JF. Pan-Asian Trauma outcomes study (PATOS): rationale and methodology of an international and multicenter trauma registry. Prehospital Emerg Care. 2017;22(1):58–83. 10.1080/10903127.2017.1347224.

### P46. Characterising paediatric critical care trauma patients

#### Anand Krishna^1^, Elaine Cole^2^

##### ^1^Royal London Hospital, Barts Health NHS Trust, UK; ^2^Centre for Trauma Sciences, Blizard Institute, Queen Mary University London, UK

*Scandinavian Journal of Trauma, Resuscitation and Emergency Medicine* 2024, **32(S1)**: P46

**Background** Trauma is the largest cause of mortality in children and young people (CYP) [1], but little is known about the role of critical care (CC) in supporting the paediatric major trauma patient.

**Methods** This retrospective study examined data from four Major Trauma Centres (MTCs) over three years in patients < 16 years. Primary outcome was CC admission and secondary outcome was CC mortality.

**Results** 269/1594 patients (16.8%) required critical care. CC patients were older (10y vs 6y, *p* < 0.001), had higher injury severity scores (ISS) (20 vs 9, *p* < 0.001), required more massive transfusion (10.8% vs 1.1%, *p* < 0.001) and early surgery (40.5% vs 18.2%, *p* < 0.001), and had higher mortality rates (7.8% vs 1.1%, *p* < 0.001). Multivariable analysis showed CC admission was associated with older age (*p* = 0.01), increasing Injury Severity Score [ISS] (*p* < 0.001), early surgery (*p* < 0.001) and TXA administration (*p* = 0.02). Mortality was associated with younger age (*p* = 0.01), increasing ISS (*p* < 0.001), massive transfusion (*p* < 0.001), and spinal injuries (*p* = 0.04) or injuries categorised as 'other' (*p* < 0.001). Deaths within CC were similarly associated with younger age (*p* = 0.02), higher ISS (*p* = 0.001) and massive transfusion (*p* = 0.02). Injury patterns and mechanisms varied between age groups, with a significant burden of traumatic brain injury in younger patients (68% of < 1, 45% 1–4yo, 20% in all other age groups, *p* < 0.001). Stabbings, vehicle collisions and falls > 2 m were all more common in older children (all *p* < 0.001).

**Conclusions** Approximately 1 in 6 major trauma CYP patients required critical care, but were more severely injured, required higher rates of emergency interventions and were older, although younger patients had higher mortality. Future analysis of age related risks is required.

**Reference**

1. Wolfe I, Macfarlane A, Donkin A, Marmot M, Viner R. Why children die: death in infants, children and young people in the UK. Royal College Of Paediatrics And Child Health, National Children’s Bureau British Association For Child And Adolescent Public Health. 2014.

### P47. Should traumatic liver injuries have routine radiological surveillance: a five-year review at the Royal London Hospital

#### Emily Long^1^, Eleanor Smith^1^, Susan Cross^2^, Elaine Cole^1^, Kate Hancorn^2^

##### ^1^Centre for Trauma Sciences, Blizard Institute, Queen Mary University of London, London, England; ^2^Royal London hospital, Barts health NHS trust, London, England

**Correspondence:** Emily Long (Emily.long@uhs.nhs.uk)

*Scandinavian Journal of Trauma, Resuscitation and Emergency Medicine* 2024, **32(S1)**: P47

**Background** The liver is the second most frequently injured solid organ in abdominal trauma [1]. Following initial management of traumatic liver injury, some patients will develop delayed complications. Routine re-imaging of patients to screen for delayed complications has been recommended by some centres although this lacks consensus. The aim of this study was to review the role of surveillance imaging in the management of liver trauma at the Royal London Hospital, a Major Trauma Centre.

**Methods** This was a single centre retrospective review of all adult patients with blunt or penetrating liver trauma presenting to the Royal London Hospital over a five-year period, from January 2016 to January 2021. All liver injuries were graded by a radiologist according to American Association for the Surgery of Trauma(AAST) score [1]. Primary outcome was rate of delayed hepatic complication.

**Results** In 378 patients with liver trauma, 290(77%) were male, the median age was 31(IQR 22–45), two-thirds had blunt injury (240, 64%) and median ISS was 25(IQR 14–41). Two-thirds (n = 245) were managed non-operatively. 137(36%) patients were re-imaged, with delayed hepatic complications reported in 38(10%). Rate of delayed complication by AAST grade was 1.5% (n = 1) for AAST-I, 0.9% (n = 1) for AAST-II, 14.2% (n = 18) for AAST-III, 25.5% (n = 13) for AAST-IV and 26.3% (n = 5) for AAST-V. Incidence of delayed complication was significantly associated with penetrating injury (*p* = 0.04), initial operative management (*p* = 0.0176) and an AAST grade of III-V (*p* < 0.0001). In the 69.3% of patients (n = 95) where re-imaging was triggered by clinical deterioration, 33% (n = 31) were found to have a delayed complication. In the remaining 30.7% re-imaged routinely, 16% (n = 7) developed a delayed complication. Following identification of delayed complications, 27(71%) patients required further intervention, of which all had an AAST grade injury of III-V.

**Conclusion **Our findings support routine surveillance imaging in patients with AAST grade III-V liver injury. Further prospective studies are required to establish the optimum time for re-imaging and appropriate modality.

**Reference**

1. Kozar RA, Crandall M, Shanmuganathan K, Zarzaur BL, Coburn M, Cribari C, Kaups K, Schuster K, Tominaga GT; AAST Patient Assessment Committee. Organ injury scaling 2018 update: Spleen, liver, and kidney. J Trauma Acute Care Surg. 2018 Dec;85(6):1119–1122.

### P48. A rise in taskings to penetrating injuries in the Midlands: a retrospective study

#### Olivia Cunningham^1^, Govind Oliver^2^, Mark Beasley^2^, Caroline Leech^2^

##### ^1^University of Birmingham Medical School, UK; ^2^The Air Ambulance Service, Rugby, UK

**Correspondence:** Govind Oliver (govind.oliver@theairambulanceservice.org.uk)

*Scandinavian Journal of Trauma, Resuscitation and Emergency Medicine* 2024, **32(S1)**: P48

**Background** Penetrating injuries have increased 40% in England and Wales since 2011. 4683(23%) of all recorded assault with a bladed article offences occurred in the Midlands [1]. The Air Ambulance Service (TAAS) provides a 24/7 critical care doctor and critical care paramedic service in this region. We aimed to understand the epidemiology of the incidents responded to by our service.

**Method** We reviewed all patients with penetrating injuries from any cause assessed by TAAS between 1st January 2018 and 31st December 2023. ‘Stand downs’ were excluded. Anonymised data were collected from the service database on patient demographics, injury, treatment and event outcome.

**Results **TAAS attended 772 patients. This increased from 75 in 2018 to 146 in 2023. Median age was 30 (range 1–94) years, 9% were children, and 86% were male. There were 123 reported self-inflicted injuries (16%), and 24 cases were noted as domestic violence (3%). 8% of patients died with 83% of these occurring on scene. Fatalities rose significantly from 4% in 2018 to 13% in 2023. Of the fatalities: 81% were male, 44% had just one wound, 22% were self-inflected, and thoracic injury was the most common site. A quarter of female fatalities were recorded as domestic violence. Resuscitative thoracotomy was performed in 31(4%) cases (average of 5 per year). Since carrying blood, pre-hospital products were administered in 11(8%) and 16 (11%) cases in 2022 and 2023 respectively. Pre-Hospital Emergency Anaesthesia was uncommon (1–3% cases per year).

**Discussion**

The number and severity of incidents has risen over the last 6 years. This may be mirrored nationally. Prehospital practitioners should consider how they can be involved in primary prevention to reduce the burden of penetrating trauma. Reporting of self-harm and domestic violence is important to better identify trends.

**Reference**

1. ONS Centre for Crime and Justice. (2024, March). *Crime in England and Wales: Police Force Area data tables*.

### P49. Pediatric Prehospital Airway Management in a German HEMS system

#### Melanie Rudolph^1^, Florian Reifferscheid^2^, Jörg Braun^2^, Marcus Rudolph^2^

##### ^1^Department of Neonatology and Pediatric Intensive Care, University Hospital Mannheim, Mannheim, Germany; ^2^DRF Stiftung Luftrettung gAG, Filderstadt, Germany

**Correspondence:** Marcus Rudolph (marcus.rudolph@drf-luftrettung.de)

*Scandinavian Journal of Trauma, Resuscitation and Emergency Medicine* 2024, **32(S1)**: P49

**Background** Airway management is crucial in prehospital emergency medicine. In pediatric emergencies a safe airway is essential. HEMS crews provide intensive care at the site of the emergency, however pediatric emergencies are uncommon, pediatric intubation is even more infrequent. Thus we evaluated the incidence and first pass success rate in a German HEMS system.

**Method** The DRF Stiftung Luftrettung gAG operates 31 helicopters all over Germany, the medical team comprises a paramedic and a doctor. There is no specialised paediatric training other than an APLS course and experience in paediatric anaesthesia. The bases deliver both primary and secondary missions. We evaluated all tasks in children younger than 14 years from 2012 up to 2021.

**Results** The DRF carried out 482,074 missions in the above-mentioned period, 31,617 (6.6%) children were treated. Endotracheal intubation was performed by the HEMS team in 924 (2.9%) cases. First pass success was achieved in 799 cases (86.5%), a second in 112 (12%), a third in 9 (1%), more than 3 attempts (0.3%) were required in 3 patients. A stylet was used as an aid in 329 cases (35.6%) and a video laryngoscope in 116 cases (12.6%); the use of a bougie was not documented.

**Conclusion** The rates of pediatric intubation were low in our collective. The first pass success was acceptable 86.5% but lower as in other systems [1, 2]. The reason may be a low number of adjunct use like a stylet or a video-laryngoscope (which was introduced in pediatric sizes in 2017). The current guideline for intubation in neonates and infants strongly recommends the use of video-laryngoscopy [3]. The bottom line is to improve the use of adjuncts as bougies or video-laryngoscopes, which requires comprehensive introduction of the devices as well as a change in training culture and adjustments in clinical governance.

**References**

1. Solan T, Cudini D, Humar M, Forsyth N, Meadley B, St. Clair T, u. a. Characteristics of paediatric pre‐hospital intubation by Intensive Care Paramedics. Emerg Medicine Australasia. Oktober 2023;35(5):754–8.
2. Burns BJ, Watterson JB, Ware S, Regan L, Reid C. Analysis of out-of-hospital pediatric intubation by an Australian helicopter emergency medical service. Ann Emerg Med. April 2017 [cited 3. Mai 2017]; Available at: http://linkinghub.elsevier.com/retrieve/pii/S0196064417303165.
3. Disma N, Asai T, Cools E, Cronin A, Engelhardt T, Fiadjoe J, u. a. Airway management in neonates and infants: European Society of Anaesthesiology and Intensive Care and British Journal of Anaesthesia joint guidelines. Br J Anaesth . November 2023;S0007091223004981.

### P50. Exploring the relationship between emotional regulation in clinicians and performance during trauma resuscitation: a systematic review

#### Omar Ahmed^1^, Jacqueline Rappoport^1^

##### ^1^Centre for Trauma Sciences, Queen Mary’s University, London, United Kingdom

**Correspondence:** Omar Ahmed (omar.ahmed6@nhs.net)

*Scandinavian Journal of Trauma, Resuscitation and Emergency Medicine* 2024, **32(S1)**: P50

**Background** Trauma resuscitation is a high pressure and time sensitive situation, carrying a high risk of patient morbidity. Effective resuscitation requires assimilating large amounts of information in a dynamic and complex environment as well as rapid decision making and team management [1]. Resuscitation places significant stress on trauma clinicians in the prehospital and hospital environments. The ability to emotionally regulate and manage the immediate stress response is a necessary skill, which facilitates the application of lifesaving technical and non-technical skills [2]. Failure to emotionally regulate leads to clinicians becoming overwhelmed and a deterioration in performance during resuscitation.

**Aims **This systematic review aims to identify emotional regulation strategies used by clinicians during trauma resuscitation, explore their efficacy, and impact on resuscitation performance.

**Methods **A systematic search was conducted across Pubmed, OVID and Google Scholar databases for all articles exploring emotional regulation strategies during real life trauma resuscitation, excluding simulation. Retrieved articles (n = 81,494) underwent title, abstract screening, and then full text review to identify the final articles (n = 5) eligible for inclusion. A narrative synthesis was undertaken as metanalysis was not possible due to heterogeneity.

**Results **A series of emotional regulation strategies were identified such as visualisation, cognitive offloading, and positive self-talk [3]. These strategies were either inward focussed such as tactical breathing exercises, or external focussed such as mental model sharing and checklist use [3]. Emotional regulation strategies enabled clinicians to feel calmer, more confident, less stressed, and may improve performance during resuscitation.

**Conclusions **Clinicians use emotional regulation strategies during trauma resuscitation. These strategies can be nurtured and developed into a teachable set of skills to optimise performance during resuscitation. Emotional regulation during resuscitation is under studied, and future research into this topic is recommended.

**References**

1. Groombridge C, Kim Y, Maini A, Smit D, Fitzgerald M. Stress and decision-making in resuscitation: a systematic review. Resuscitation. 2019;114:115–22.
2. LeBlanc VR. The effects of acute stress on performance: implications for health professions education. Acad Med. 2009;84(10):S25–S25.
3. Groombridge CJ, Maini A, Ayton D, Soh SE, Walsham N, Kim Y, et al. Emergency physicians’ experience of stress during resuscitation and strategies for mitigating the effects of stress on performance. Emerg Med J. 2021; Available from: https://emj.bmj.com/content/early/2021/12/13/emermed-2021-211280.

### P51. Changes in patient demography, fracture complexity and surgical management of proximal tibial fractures over 2 decades from an interrupted time series analysis—the need for greater systemwide vigilance and better documentation.

#### Hannah Noone^1^, Paul Stanier^1^, Vinayak Venugopal^1^, Adam Brooks^1^, Sunny Deo^1^

##### ^1^Department of Trauma and Orthopaedics, Great Western Hospital NHSFT, Swindon, UK

**Correspondence:** Correspondence (Vinayak.Venugopal@nhs.net)

*Scandinavian Journal of Trauma, Resuscitation and Emergency Medicine* 2024, **32(S1)**: P51

**Background** Open reduction internal fixation has remained the standard for majority of displaced intra-articular proximal tibia fractures. Our aim was to analyse changes in demography, injury types, method of fixation and early outcomes over a 2-decade timespan.

**Methods **A single Centre, retrospective analysis of patients having surgery for closed intra-articular fractures of the proximal tibia AO/OTA 41A-C over two 5-year time periods from 2004 to 2008 and 2014 to 2018 was undertaken. Patients’ age, sex, injury mechanism, the presence of other significant injuries, comorbidities, fixation method undertaken, time to definitive surgery and early outcomes relating to length of stay and early post-operative complications 42 days from index surgery were noted.

**Results **There were 31 patients. Over the timespan there were notable changes in demography, fracture types and modes of fixation. Demographics showed an increasing mean age from 43 to 49 years, increased female proportion (40% Vs 57%), higher proportion with major comorbidities (10% Vs 55%),reduced rate of high energy injuries (50% Vs 29%) but more complex fracture patterns highlighted by the increase proportion of AO/OTA C2 and C3 fractures in the latter group (20% Vs 38%), resulting in increased double and triple plate constructs from 10 to 50%.Despite these, the time to surgery reduced from 4.6 to 3.9 days and mean length of hospital stays reduced from 11.7 to 8.1 days.

**Conclusions** This study confirms trends towards greater fracture complexity and represents a greater burden in terms of pre-operative planning, surgical time and skillset requirements, with implications for treatment costs. These issues are unlikely to be known to patients, healthcare practitioners, health care commissioners or the wider healthcare system. There is no clear reason for increasing fracture complexities in terms of displacement and fragmentation and whether this an ongoing trend. Better on-going observation, larger similar studies and better documentation of such fractures is recommended.

### P52. Video laryngoscopy vs. direct laryngoscopy in a UK pre-hospital physician/critical care paramedic Helicopter Emergency Medical Service

#### Adam J. R. Watson^1^, Julian Hannah^2^, Oluwasemire Adetoro^3^, James O.M Plumb^1,2,4^

##### ^1^University Hospital Southampton NHS Foundation Trust, Southampton, United Kingdom; ^2^Hampshire and Isle of Wight Air Ambulance, Southampton, United Kingdom; ^3^University of Southampton Medical School, Southampton, United Kingdom; ^4^ Perioperative and Critical Care Theme, NIHR Southampton Biomedical Research Centre, University Hospital Southampton NHS Foundation Trust, Southampton, United Kingdom and Integrative Physiology and Critical Illness Group, Clinical and Experimental Sciences, Faculty of Medicine, University of Southampton, Southampton, United Kingdom

**Correspondence:** James O.M Plumb (plumb@soton.ac.uk)

*Scandinavian Journal of Trauma, Resuscitation and Emergency Medicine* 2024, **32(S1)**: P52

**Background **It is recognised that multiple attempts at intubation are associated with harm. However, it remains unclear whether video laryngoscopy (VL) significantly improves pre-hospital endotracheal intubation success compared to direct laryngoscopy (DL) in critically ill patients. While operating theatre studies strongly favour VL, some pre-hospital studies suggest it may worsen outcomes [1–3].

**Methods **This single-centre retrospective service evaluation included consecutive critically ill patients requiring pre-hospital endotracheal intubation by Hampshire & Isle of Wight Air Ambulance between 1st November 2018 and 22nd April 2024. This time period saw the introduction of VL with the *option* to use it. Collected data encompassed patient demographics, intubation indication, induction drugs, and intubation technique (type of laryngoscopy, grade of view, number of attempts, and complications). The primary outcome was first-pass success, comparing VL and DL groups, with significance set at *p* = < 0.05.

**Results **We included 1281 patients (median age 56, 69% male), of whom 478 (37%) received VL and 803 (63%) received DL. The most common intubation indications were major trauma (n = 347, 27%), medical cardiac arrest during CPR (n = 341, 27%), and cardiac arrest post-ROSC (n = 279, 22%). First-pass success was 93% (n = 443) in the VL group and 83% (n = 670) in the DL group, with an absolute risk difference of 9.2% (95% CI 5.8–12.7%). Since the introduction of VL in June 2022, both the proportion of VL intubations and first-pass success rates have increased annually.

**Conclusion **Our findings support the routine use of VL for pre-hospital endotracheal intubation.

**References**

1. Tim M et al. Has the time really come for universal videolaryngoscopy? Cook. Br J Anaesthe. 129(4):474–477.
2. Jiang J, Ma D, Li B, Yue Y, Xue F. Video laryngoscopy does not improve the intubation outcomes in emergency and critical patients—a systematic review and meta-analysis of randomized controlled trials. Crit Care. 2017 Nov 24;21(1):288. 10.1186/s13054-017-1885-9. PMID: 29,178,953; PMCID: PMC5702235.
3. Pourmand A, Terrebonne E, Gerber S, Shipley J, Tran QK. Efficacy of Video Laryngoscopy versus Direct Laryngoscopy in the prehospital setting: a systematic review and meta-analysis. Prehospital Disast Med. 2023;38(1):111–121. 10.1017/S1049023X22002254.

### P53. SAR Missions In The Barents sea—characteristics of missions performed

#### Silje Aune^1^, Lasse Raatiniemi^2^, Torben Wisborg^1^

##### ^1^Emergency Medicine Department, Finnmark Hospital, Hammerfest, Norway; ^2^Surgical & intensive care unit and air ambulance department, University Hospital of North Norway, Tromsø, Norway

**Correspondence:** Silje Aune (silje.ingeborg.aune@finnmarkssykehuset.no)

*Scandinavian Journal of Trauma, Resuscitation and Emergency Medicine* 2024, **32(S1)**: P53

**Background** The Barents Sea presents significant challenges for search and rescue operations due to its remote areas, long distances, harsh weather, and seasonal darkness. The Norwegian 330 Squadron’s rescue helicopters, stationed in Banak manage search and rescue missions as well as air ambulance operations in this region. Previous studies, such as Haagensen et al. [1], have explored operational conditions and medical outcomes for Barents Sea missions. Our study updates and expands this knowledge by analyzing missions from 2000 to 2022.

**Method **We conducted a retrospective, quantitative analysis of air ambulance missions performed by the 330 Squadron Banak from January 1, 2000, to December 31, 2022. Data were sourced from the Norwegian Air Ambulance Service’s database (LABAS). All air ambulance requests in the Barents Sea during this period were included.

**Results** Out of 415 requests, 133 were declined or cancelled due to lack of need (88), coordination with other resources (29), or weather (7). 282 missions were completed. The patients included 272 men and 10 women, with an average age of 41. Nationalities were 47% Norwegian, 35% Russian, and the rest other nationalities. Average response time was 187 min, transport time 93 min, and total mission time 362 min. Medical conditions included gastrointestinal disorders (62), cardiovascular diseases (45), and neurological conditions (22). Injuries included upper extremities (40), lower extremities (24), and head injuries (23). NACA scores showed 155 patients with score 3 and 81 with score 4. Analgesics, antibiotics, and cardiovascular drugs were commonly administered.

**Discussion **This study shows that rescue missions in the Barents Sea mainly involve working-age men with gastrointestinal and cardiovascular conditions, as well as crush injuries and falls. NACA scores indicate severe but non-life-threatening conditions. These findings can enhance resource planning, training, and preparedness for future Arctic rescue missions.

**Reference**

1. Haagensen R, Sjøborg KA, Rossing A, Ingilae H, Markengbakken L, Steen PA. Long-Range rescue helicopter missions in the Arctic. Prehosp Disaster Med. 2004;19(2):158–63. 10.1017/s1049023x00001679. PMID: 15,508,199.

### P54. Cardiopulmonary resuscitation induced consciousness: prevalence, prognosis and management

#### Jessica R Webb^1^, Lisa Ramage^1^

##### ^1^School of Clinical Medicine, University of Cambridge, Cambridge, UK

**Correspondece:** Jessica R Webb (jrw205@cam.ac.uk)

*Scandinavian Journal of Trauma, Resuscitation and Emergency Medicine* 2024, **32(S1)**: P54

**Background** Cardiopulmonary resuscitation induced consciousness (CPRIC) is a phenomenon in which patients either exhibit or are later found to demonstrate some level of consciousness while receiving cardiopulmonary resuscitation (CPR) during cardiac arrest. This review aims to synthesise the evidence from case reports, observational studies and reviews on the incidence and characteristics of CPRIC, the characteristics of the patients affected, the impact of CPRIC on patients and healthcare workers and how CPRIC should be managed.

**Method** Articles were identified using a MEDLINE search for keywords, MeSH terms and multipurpose terms ‘cardiopulmonary resuscitation’, ‘awareness’, ‘consciousness’, ‘responsiveness’, ‘CPR-induced consciousness’, ‘CPR induced consciousness’, ‘cardiopulmonary resuscitation-induced consciousness’ ‘cardiopulmonary resuscitation induced consciousness’ and ‘CPRIC’. The results and their reference lists were assessed for relevance.

**Results** A total of 45 articles were selected. This included 28 case reports including 35 patients, 11 observational studies including 6 patient studies and 5 rescuer studies, and 6 review articles. The prevalence of CPRIC is reported to be between 0.23 and 0.9% and between 42.1 and 91% of practitioners that regularly attended cardiac arrests report to have witnessed CPRIC. Several patient and situational factors were found to be associated with CPRIC, and CPRIC was associated with a higher likelihood of survival. A wide variety of strategies to manage cases of CPRIC were found.

**Conclusion** CPRIC is uncommon but increasingly reported. A standardised definition of CPRIC is required to facilitate standardised reporting. Practitioners should be made aware that while it can be a stressful presentation to manage, it is associated with increased likelihood of survival. A consensus guideline on management is required, likely including ketamine and midazolam.

### P55. Development of an Enhanced resuscitation course—proof of concept

#### John Ramage^1^, Sean McKeon^2^, James Raitt^3^

##### ^1^University of Hertfordshire, UK; ^2^Frimley Park Hospital, UK; ^3^Thames Valley Air Ambulance, Stokenchurch, UK

*Scandinavian Journal of Trauma, Resuscitation and Emergency Medicine* 2024, **32(S1)**: P55

**Background **The Resuscitation Council UK Advanced Life Support course provides a framework and shared common language for clinicians to approach cardiac arrest, but there remains a gap for senior clinicians to be trained in higher level technical and teamwork skills. The Enhanced Resuscitation (ER) course aimed to address this gap, we report on the content and feedback from the first one day course.

**Methods **The ER course was run over one day in the St George's University simulation suite with a faculty of 5 consultants, 2 senior registrars and a critical care outreach nurse. The candidates were 7 registrars and 4 nurses. The format involved pre-course reading, workshops on technical skills (mechanical CPR, ultrasound and surgical procedures) and simulations with extended time for debrief and discussion. Scenarios included refractory VF, asthmatic arrest, traumatic arrest and maternal arrest.

**Results **All candidates graded the course maximally relevant to their clinical practice. When asked as to what extent it would improve their management of cardiac arrest, 87.5% of respondents rated it 5/5. Written feedback centred on the usefulness of simulated complex scenarios and decision-making in 'grey areas' beyond the remit of ALS, consolidated by the opportunity for detailed reflection with expert faculty. A focus on 'human factors' and leadership in prolonged resuscitation were similarly well received. All candidates agreed the course was sufficiently beneficial to their development that they would fund their attendance at a future course personally or from their study budget.

**Conclusions **We have shown that it is feasible to deliver a high-fidelity enhanced resuscitation course, focusing on technical and non-technical skills beyond what is offered in ALS, successful delivery depends on a credible faculty who are able to facilitate high-fidelity simulation and debrief. The positive feedback and enthusiasm from candidates show the desire for an enhanced life support course.

### P56. Advanced development of radiological and nuclear medical countermeasures at the biomedical advanced research and development authority (BARDA)

#### Julie Bergmann^1^, Andrew Cap^1^, John Esker^1^, Andrew Haskell^1^, Corey Hoffman^1^, William Hoots^1^, Juanita Jones^1^, Brian Moyer^1^, Richard Moreno^1^, Toby Silverman^1^, Dana Tedesco^1^, Beryl Voigt^1^, and Mary Homer^1^

##### ^1^Division of Chemical, Biological, Radiological, and Nuclear Countermeasures (CBRN), Biomedical Advanced Research and Development Authority (BARDA), Administration for Strategic Preparedness and Response (ASPR), U.S. Department of Health and Human Services. Washington, DC, USA

**Correspondence:** Julie Bergmann (Julie.Bergmann@hhs.gov)

*Scandinavian Journal of Trauma, Resuscitation and Emergency Medicine* 2024, **32(S1)**: P56

The mission of the Biomedical Advanced Research and Development Authority (BARDA), part of the U.S. Department of Health and Human Services (HHS), is to develop medical countermeasures (MCMs) that address the public health and medical consequences of chemical, biological, radiological, and nuclear (CBRN) accidents, incidents, and attacks; pandemic influenza; and emerging infectious diseases.

For radiological and nuclear (RN) threats, HHS focuses on nuclear detonation as a consensus scenario for preparedness, response, and recovery planning. Because the medical consequences of a nuclear detonation are more expansive and complex than acute radiation syndrome alone, BARDA’s RN Medical Countermeasures Program actively works to develop MCMs for hemorrhage, trauma, and other anticipated injuries. Current RN Program initiatives focus on developing next generation blood products, enabling technologies (e.g., organ-on-chip), and establishing collaborations to support improved understandings of disease/injury progression. For all products, BARDA prioritizes solutions that address multiple threat areas, are repurposed technologies already approved for use, and enable use at the most appropriate time point along the continuum of care to allow for maximal therapeutic potential.

BARDA recognizes that during a CBRN response, access to definitive health care resources during resource or time limited scenarios will be necessary. BARDA knows that engaging the emergency medical community in its MCM developmental efforts is imperative to understanding end-user needs and familiarizing the emergency medical community with available and upcoming products. As such, BARDA is actively working with and seeking input from end-users in the pre-hospital and emergency services spaces.

This presentation will focus on BARDA as an advanced development partner of MCMs that address injuries resulting from trauma. The presentation will also highlight ongoing efforts to engage the end-user community (emergency medical personnel) to identify gaps in the development pipeline. A complete look at opportunities with BARDA can be found at www.medicalcountermeasures.gov.

### P57. The use and impact of regional analgesia after traumatic rib fractures: a multicentre retrospective study

#### R A Bradley^1^, G Manoharan^2^, C Blatchford^1^, A Hodson^3^, J Organ^4^, D Bew^2^, M Marsden^2,5^

##### ^1^Queen Elizabeth Hospital, Lewisham and Greenwich NHS Trust, ^2^Kings College Hospital, Kings College NHS Trust, ^3^University Hospital Lewisham, Lewisham and Greenwich NHS Trust, ^4^Department of Anaesthesia, Royal London Hospital, Bart’s Health NHS Trust, ^5^Academic Department of Military Surgery and Trauma, Research and Clinical Innovation, Ministry of Defence

**Correspondence:** Miss Rebecca A Bradley (rebecca.bradley16@nhs.net)

*Scandinavian Journal of Trauma, Resuscitation and Emergency Medicine* 2024, **32(S1)**: P57

**Background** Rib fractures, prevalent in trauma patients, are linked to complications, longer hospital stays, and mortality. While regional analgesia (RA) improves pain, its clinical impact remains unclear. We assessed the association between RA and patient outcomes (length of stay (LOS), pulmonary complications, mortality) and the types of RA used in adult patients with traumatic rib fractures. We also aimed to assess differences between Major Trauma Centres (MTC) and Trauma Units (TU).

**Methods** A multicentre retrospective study was undertaken in adult patients admitted with traumatic rib fractures in three hospitals within one Major Trauma Network. A convenience sample of patients were included admitted over two years from 01.01.2021. Multivariate regression analysis identified factors related to LOS, pulmonary complications and mortality.

**Results** Of the 427 patients (median age 66 years (range 18–99), 67% male), increasing STUMBL Score and chest drain use were associated with increased LOS. ESP catheters were the most commonly use form of RA. Adjusted analysis showed RA correlated with a reduced LOS of 7.9 days (95% CI 2.5–13.3, *p* < 0.001) but an increased risk of pulmonary complications (OR 3.96, 95% CI 2.168–7.326, *p* < 0.001). RA did not affect mortality. Patients treated at MTCs had a higher prevalence of pneumothorax (*p* = 0.011) and lung contusions (*p* < 0.001) compared to TU patients, but overall outcomes were similar.

**Conclusions** In patients with traumatic rib fractures, regional analgesia correlated with shorter hospital stays, but not reduced mortality. Further investigation through robust trials is warranted to definitively establish RA's impact.

### P58. Feasibility of electronic patient reported outcome measures (PROM) in trauma patients

#### Ingri Grimnes Olsen^1,*^, Andrew Garratt^2^, Astrid Woodhouse^3^, Mona Stedenfeldt^1^, Torunn Hatlen Nost^4^, Olav Roise^5^, Tom-Ivar Lund Nilsen^6^, Oddvar Uleberg^7^

##### ^1^National Quality and Competence Network for Complex Pain Conditions, St. Olavs Hospital, Trondheim University Hospital, Norway; ^2^National Institute of Health, Oslo, Norway; ^3^Department of Circulation and Medical Imaging, Norwegian University of Science and Technology, Trondheim, Norway; ^4^Department of Mental Health, Norwegian University of Science and Technology, Trondheim, Norway; ^5^Department of Public Health and Nursing, Norwegian University of Science and Technology, Trondheim, Norway; ^6^University of Oslo, Norway; ^7^Department of Emergency Medicine and Pre-hospital services, St. Olav’s Hospital, Trondheim University Hospital, Norway

**Correspondence:** Ingri Grimnes Olsen (ingri.grimnes.olsen@stolav.no)

*Scandinavian Journal of Trauma, Resuscitation and Emergency Medicine* 2024, **32(S1)**: P58

**Background** Trauma is a major cause of long-term morbidity and disability. Survivors of trauma may experience negative physical, socio-economic and quality of life related effects years after injury. Early identification of vulnerable patients can help improve tailored care to enhance health and quality of life. PROMs information can be used to identify such patients and monitor their changes in health over time. The PROMIS-29 is a modern short-form health profile that assesses mental, physical and social health across several domains. This study aims to assess feasibility and response rate (RR) of PROMIS-29 as an electronic PROM in trauma patients.

**Methods**

 rospective study including patients > 18 years admitted by a multidisciplinary trauma team at St. Olavs University Hospital from 01.09.21 to 31.10.22. Consenting patients were to complete PROMIS-29 at baseline, after 14 days and after 3, 6 and 12 months post-injury. Baseline was completed at the hospital or by smartphone following discharge. Follow-up was completed electronically.

**Results **196 patients were included. The mean age was 51 years and 34% were women. All patients completed the baseline measurement. At follow-up, the RR was 59% at 14 days, 49% at 3 months, 47% at 6 months, and 41% at 12 months.

**Conclusion/Discussion **The largest reduction occurred between baseline and 14 days post-injury, when patients completed PROMIS-29 electronically. PROMs information is necessary to inform patient care but the downward trend of RRs as seen here and in cohort and registry-based studies, suggests suboptimal methods of data collection. Reductions in RR may stem from patients having problems with the electronic solution, or them being less motivated to respond over time when their reporting does not affect their care plan. Therefore, a follow-up study is planned which will include trauma patients completing PROMIS-29, alongside qualitative interviews. Based on the responses to PROMIS-29, tailored follow-up by a rehabilitation specialist will be offered.

### P59. (Re-) Defining the frequency of major trauma in England

#### Edward C Crosbie^1^, Christopher Aylwin^2^

##### ^1^MSc Trauma Sciences, Queen Mary University London, London, UK; ^2^Centre for Trauma Sciences, Blizard Institute, Queen Mary University London, London, UK

**Correspondence:** Edward C Crosbie (ha211642@qmul.ac.uk)

*Scandinavian Journal of Trauma, Resuscitation and Emergency Medicine* 2024, **32(S1)**: P59

**Background** In 2010, the National Audit Office published the statistic of ‘20,000 major trauma cases per annum’ that has become dogma in trauma education [1]. Major trauma is the leading cause of death under forty, and current service delivery guidelines state candidate patients should bypass trauma units for direct conveyance to major trauma centres [2]. It is hypothesised that the current frequency exceeds the historical figure of 20,000. This contemporary research aimed to estimate the frequency and distribution of major trauma patients in England from 2021 to 2022 from published NHS Digital data, reporting 22,839,832 emergency department attendances, creating an estimated frequency of 45,680 from a predicted threshold of 0.2% [3].

**Methods** Clinicians working in major trauma service delivery networks participated in a non-probability sampling survey, subjectively identifying major trauma from 205 closed questions of anatomical trauma descriptions. Data from 103 participants was used to calculate the frequency of consensus candidate major trauma compared to NHS Digital A&E attendance frequency. Distribution was assessed within the NHS trusts that contain England’s twenty-seven major trauma centres.

**Results** The study’s findings recognise that major trauma is a significant prehospital disease, with a modern frequency in England that exceeds 112,842 cases per year, suggesting that major trauma is highly under-represented in current literature and trauma education.

**Conclusions** Major trauma can subjectively be identified by anatomical distribution: Injury patterns were divided between torso trauma (49%) and head trauma, including TBI, neck and spinal cord (44%). Appendicular (limb) major trauma, excluding the pelvis, comprises only 7%. Survey results suggest a large discrepancy in how trauma cases are identified in prehospital settings, with 72% of candidate major trauma patients being treated in trauma units and not conveyed directly to major trauma centres, however, it is recognised that not all candidate major trauma patients will need conveyance to major trauma centres.

**References**

1. Comptroller and Auditor General. Major Trauma Care in England. National Audit Office. HC 213, 2010.
2. National Institute for Health & Care Excellence (NICE), Major Trauma Service Delivery: NICE Guideline [NG40]. 2016.
3. NHS England. Hospital Accident & Emergency Activity 2021-22 Official Statistics. NHS Digital. 2022.

### P60. Pilot comparison of aortic versus Intravenous Infusion of hemostatic agents in a swine model of noncompressible truncal haemorrhage

#### Nathan J. White^1^, Xu Wang^1^, Kristyn Ringgold^1^, Lauren Neidig^1^, Chang Yeop Han^1^, Trey Pichon^2^, Ethan Mickelson^2^, Trevor Corrigan^2^, Suzie Pun 2, Susan Stern 1

##### ^1^Department of Emergency Medicine, University of Washington, Seattle, WA, USA; ^2^Department of Bioengineering, University of Washington, Seattle, WA, USA

**Correspondence:** Nathan J. White (whiten4@uw.edu)

*Scandinavian Journal of Trauma, Resuscitation and Emergency Medicine* 2024, **32(S1)**: P60

**Background** Noncompressible truncal hemorrhage (NCTH) is a leading cause of death after injury. Arterial NCTH is particularly difficult to manage, oftentimes requiring aortic occlusion maneuvers. Intravenous hemostats such as tranexamic acid (TXA) can confer a survival benefit after trauma, but their optimal use during NCTH remains unclear. We hypothesize that direct delivery of hemostats into the aorta may promote more effective hemostasis of NCTH by delivering a greater concentration of hemostatic agent directly to the internal wound site. To evaluate this hypothesis, we compared the effects of aortic versus intravenous infusion of TXA in a swine model of NCTH.

**Methods** We used a swine NCTH model with free internal bleeding from a 5mm infrarenal aortic tear. We randomized animals to receive 15mg/kg TXA infused either intravenously or through a small catheter placed in zone 1 of the aorta. Shed whole blood and Ringer’s Lactate solution were infused for resuscitation for up to 3 hours after injury. We compared vital signs, internal blood loss, markers of metabolic shock, and survival between groups using repeated measure ANOVA, T-test, and Kaplan Meijer survival analysis.

**Results** Aortic infusion of TXA (N = 11) demonstrated a significantly decreased mean (SD) rate of internal blood loss at 0.26 (0.31) ml/kg/min compared to intravenous infusion (N = 6) at 0.70 (0.46) ml/kg/min (*p *= 0.034). During resuscitation, aortic infusion of TXA significantly increased mean arterial blood pressure (rmANOVA *p *= 0.024) and significantly decreased arterial lactate concentration (rmANOVA *p *= 0.01) when compared to intravenous infusion. Kaplan Meijer time to event survival significantly increased with aortic infusion (KM LR p=0.008).

**Conclusion** Our pilot findings suggest that aortic infusion of hemostatic agents directly into the aorta may reduce internal bleeding and improve survival for NCTH. Further experiments are needed to achieve adequate statistical power and confirm these results.

